# Kinesin superfamily protein Kif26b links Wnt5a-Ror signaling to the control of cell and tissue behaviors in vertebrates

**DOI:** 10.7554/eLife.26509

**Published:** 2017-09-08

**Authors:** Michael W Susman, Edith P Karuna, Ryan C Kunz, Taranjit S Gujral, Andrea V Cantú, Shannon S Choi, Brigette Y Jong, Kyoko Okada, Michael K Scales, Jennie Hum, Linda S Hu, Marc W Kirschner, Ryuichi Nishinakamura, Soichiro Yamada, Diana J Laird, Li-En Jao, Steven P Gygi, Michael E Greenberg, Hsin-Yi Henry Ho

**Affiliations:** 1 Department of NeurobiologyHarvard Medical SchoolBostonUnited States; 2 Department of Cell Biology and Human AnatomyUniversity of California, Davis School of MedicineDavisUnited States; 3 Department of Cell BiologyHarvard Medical SchoolBostonUnited States; 4 Department of Systems BiologyHarvard Medical SchoolBostonUnited States; 5 Division of Human BiologyFred Hutchinson Cancer Research CenterSeattleUnited States; 6 Department of Obstetrics, Gynecology and Reproductive SciencesCenter for Reproductive Sciences, Eli and Edythe Broad Center of Regeneration Medicine and Stem Cell Research, University of CaliforniaSan FranciscoUnited States; 7 Department of Kidney DevelopmentInstitute of Molecular Embryology and Genetics, Kumamoto UniversityKumamotoJapan; 8 Department of Biomedical EngineeringUniversity of CaliforniaDavisUnited States; Johns Hopkins University School of MedicineUnited States

**Keywords:** noncanonical Wnt signaling, Ror, signal transduction, tissue morphogenesis, regulated proteolysis, Kif26b, Mouse

## Abstract

Wnt5a-Ror signaling constitutes a developmental pathway crucial for embryonic tissue morphogenesis, reproduction and adult tissue regeneration, yet the molecular mechanisms by which the Wnt5a-Ror pathway mediates these processes are largely unknown. Using a proteomic screen, we identify the kinesin superfamily protein Kif26b as a downstream target of the Wnt5a-Ror pathway. Wnt5a-Ror, through a process independent of the canonical Wnt/β-catenin-dependent pathway, regulates the cellular stability of Kif26b by inducing its degradation via the ubiquitin-proteasome system. Through this mechanism, Kif26b modulates the migratory behavior of cultured mesenchymal cells in a Wnt5a-dependent manner. Genetic perturbation of Kif26b function in vivo caused embryonic axis malformations and depletion of primordial germ cells in the developing gonad, two phenotypes characteristic of disrupted Wnt5a-Ror signaling. These findings indicate that Kif26b links Wnt5a-Ror signaling to the control of morphogenetic cell and tissue behaviors in vertebrates and reveal a new role for regulated proteolysis in noncanonical Wnt5a-Ror signal transduction.

## Introduction

The Wnt family of extracellular signaling factors orchestrates diverse developmental processes during both embryogenesis and adult tissue homeostasis. Dysfunction of Wnt signaling has been implicated in many human diseases ranging from congenital birth defects to neoplasia (Clevers and Nusse, 2012; Kikuchi et al., 2012). Wnt ligands achieve high functional versatility in part by activating multiple biochemically distinct pathways to regulate diverse cell biological processes (Veeman et al., 2003; Semenov et al., 2007).

Unlike the well-characterized ‘canonical’ Wnt pathway that signals through the transcription co-regulator β-catenin to induce target gene expression, a subset of Wnts signal independently of this Wnt/β-catenin pathway via ‘noncanonical’ mechanisms to control cytoskeleton-driven morphogenetic events such as directed cell movements, changes in cell polarization and cell adhesion (Moon et al., 1993; Veeman et al., 2003). The best-studied noncanonical Wnt, Wnt5a, is critical for the proper morphogenesis of many tissues throughout the developing embryo (Moon et al., 1993; Yamaguchi et al., 1999; Bodmer et al., 2009; Laird et al., 2011; Chawengsaksophak et al., 2012; Miyoshi et al., 2012; Cha et al., 2014). Dysregulation of Wnt5a signaling leads to birth defects and is associated with multiple pathological processes in the adult including cancer metastasis and inflammatory diseases such as atherosclerosis (Kurayoshi et al., 2006; Da Forno et al., 2008; Yamamoto et al., 2010; Jin et al., 2012; Lu et al., 2012; Bhatt and Malgor, 2014; Ford et al., 2014; Lin et al., 2014; Lu et al., 2014; Qin et al., 2015).

A growing consensus suggests that the Ror family of receptor tyrosine kinases mediates Wnt5a-dependent morphogenetic functions in the developing animal (Oishi et al., 2003; Green et al., 2008a; Mikels et al., 2009; Ho et al., 2012). However, how Wnt5a signaling via Ror receptors affects downstream cellular processes remains poorly understood. In a previous study, we found that among the many biochemical activities previously proposed to be downstream of Wnt5a signaling, only the phosphorylation of the cytoplasmic scaffolding protein Dishevelled (Dvl) required the expression of both Wnt5a and Ror proteins (Ho et al., 2012). This finding suggested that Wnt5a-Ror-dependent phosphorylation of Dvl specifically mediates the biological functions of Wnt5a signaling and led us to propose that Ror and Dvl are key components of the noncanonical Wnt5a pathway. The assignment of these proteins to a common pathway is further supported by the observation that human mutations in *WNT5A*, *ROR2*, *DVL1 and DVL3* can cause Robinow syndrome, a congenital disorder characterized by short-limbed dwarfism and morphological defects in craniofacial and genital structures, demonstrating that the Wnt5a-Ror-Dvl pathway regulates morphogenesis during human development (Afzal et al., 2000; van Bokhoven et al., 2000; Person et al., 2010; Bunn et al., 2015; White et al., 2015, 2016). However, since the function of Dvl phosphorylation is not clear, and Dvl is a common component of several signaling pathways including the canonical Wnt signaling pathway and the planar cell polarity (PCP) pathway, how the Wnt5a-Ror pathway signals to carry out its biological functions remains incompletely understood.

In this study, we conducted a whole phosphoproteome-scale mass spectrometric screen comparing wild-type cells with cells lacking the Ror family of proteins in an effort to identify additional effectors of Wnt5a-Ror signaling. The screen identified a number of candidate proteins whose levels or phosphorylation status was influenced by Wnt5a-Ror signaling, including factors involved in cytoskeletal regulation and cell adhesion, processes crucial for the morphogenesis of tissues. We then focused the remainder of the study on characterizing Kif26b, a member of the kinesin microtubule motor superfamily, which stood out as a particularly promising target of Wnt5a-Ror signaling for the following reasons. Mutations in the *C. elegans* orthologs of *Ror* and *Kif26b* produce similar neuronal migration and axon guidance phenotypes, suggesting that these molecules might function in a common molecular pathway (Wightman et al., 1996; Forrester et al., 1998). Moreover, recent studies demonstrated that Kif26b plays crucial roles in regulating cytoskeleton-driven processes, including cell migration, polarization and adhesion, raising the possibility that Kif26b could function specifically as a cytoskeletal effector of the Wnt5a-Ror pathway (Uchiyama et al., 2010; Guillabert-Gourgues et al., 2016).

Through a series of biochemical studies, we demonstrate that Wnt5a-Ror signaling regulates the steady-state abundance of Kif26b in cells via a mechanism involving the ubiquitin-proteasome system that is independent of the canonical Wnt/β-catenin-dependent pathway. Importantly, gain- and loss-of-function experiments in cultured mesenchymal cells indicate that Wnt5a-Ror-Kif26b signaling modulates mesenchymal cell migration. We also find that perturbation of Kif26b function disrupts a number of Wnt5a/Ror-dependent processes in vivo. For example, in developing zebrafish embryos, mis-expression of Kif26b causes axis and craniofacial malformations, thus mirroring the effects of mis-expression of Wnt5a or Ror in zebrafish. In developing mouse embryos, Kif26b expression is required for primordial germ cells to populate the developing gonad, a process that also requires the expression of Wnt5a or Ror proteins. Taken together, these findings establish Kif26b as a downstream effector of the noncanonical Wnt5a-Ror pathway that regulates cell and tissue behaviors during development.

## Results

### A phosphoproteomic screen identifies Wnt5a-Ror signaling targets

We sought to discover downstream effectors of Wnt5a-Ror signaling, as these could provide insight into the biochemical regulation and cell biological functions of the pathway. We reasoned that perturbation of upstream pathway components, such as the Ror receptors, would result in alterations in the biochemical regulation of downstream effectors. To test this hypothesis, we took advantage of primary mouse embryonic fibroblasts (MEFs) carrying conditional knockout alleles for the *Ror1* and *Ror2* genes (Ho et al., 2012) and screened for biochemical changes that occur upon genetic ablation of these genes. We previously showed that embryonic day 12.5 (E12.5) MEFs are a useful physiologically-relevant system for studying Wnt5a-Ror signaling. Not only are these cells derived from mesenchymal tissues that undergo active Wnt5a-Ror signaling in vivo, they continue to express high levels of endogenous Wnt5a, Ror1, Ror2 and Dvl proteins in culture and undergo autocrine/paracrine Wnt5a-Ror signaling without the addition of exogenous Wnt5a (Ho et al., 2012).

Using these conditional knockout MEFs, we performed a phosphoproteome-wide mass spectrometric screen to identify Ror-dependent changes in protein phosphorylation and/or abundance. Our reasoning was that since Wnt5a signaling regulates the phosphorylation state of known downstream components of the Wnt5a-Ror pathway, including Ror1, Ror2 and Dvl proteins (Bryja et al., 2007b; Nishita et al., 2010; Grumolato et al., 2010; Ho et al., 2012) and microarray analysis of primary MEFs lacking both Ror1 and Ror2 proteins failed to identify transcriptional changes relative to wild-type cells, Wnt5a-Ror signaling likely affects cellular functions via a transcription-independent process in MEFs (M.W.S., M.E.G., H.H.H. unpublished data).

To conduct the screen, we employed tandem mass tag (TMT) technology that enables the characterization and quantification of peptides from six experimental conditions in a single, multiplexed mass spectrometric (MS) analysis (Ting et al., 2011). This paradigm enabled the direct comparison of the identity, abundance and post-translational modifications of proteins present in cells in which the Ror proteins have been knocked out relative to wild-type control cells. Specifically, we analyzed phosphopeptides isolated from MEFs derived from E12.5 *Ror1^f/f^; Ror2^f/f^; CAG-CreER* embryos. As we described previously, the *Ror1* and *Ror2* conditional knockout alleles combined with the 4-hydroxytamoxifen (4-OHT) inducible *CAG-CreER* allele enable the acute elimination of Ror1 and Ror2 protein expression in vitro (Ho et al., 2012).

**Figure 1.**
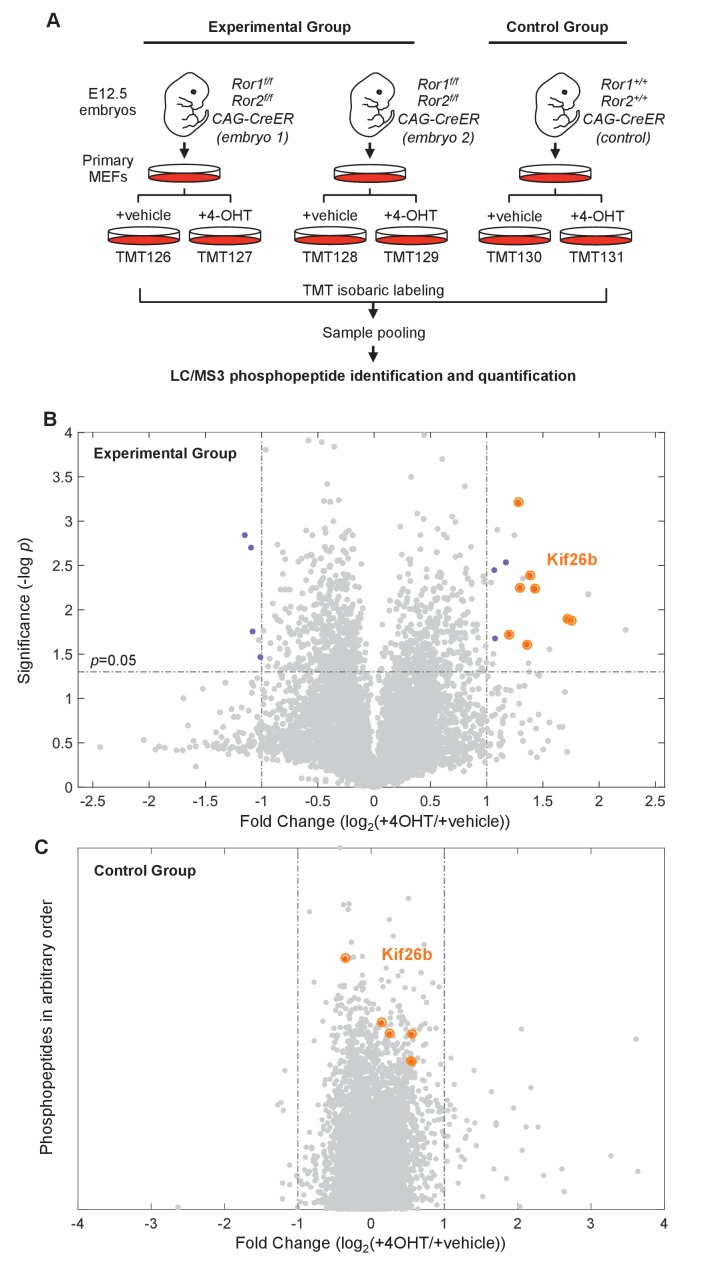
Phosphoproteomic screen identifies putative targets of the Wnt5a-Ror pathway. (**A**) Experimental design of the six-plex mass spectrometry analysis to screen for Ror-dependent biochemical cellular changes (see the main text for details of the experimental design). (**B**) Volcano plot of all phosphopeptides identified and quantified in the experimental group. Significance (y-axis) is plotted against the average fold change (4-OHT/vehicle treated samples) of the two biological replicates (x-axis). Significance values were generated with a two-sample t-test comparing values from the biological replicates. 6498 phosphopeptides were screened in each experiment. ‘Hit’ phosphopeptides, as defined in the text, are marked in blue. All Kif26b phosphopeptides are circled in orange. (**C**) Volcano plot of all phosphopeptides identified and quantified in the control group. The fold change (4-OHT/vehicle treated samples) is plotted along the x-axis. The position of the phosphopeptides along the y-axis is arbitrary, since there is only one replicate of the control group and no significance value is calculated. Kif26b phosphopeptides are circled in orange.

For the first four of the six experimental conditions analyzed in the MS screen, we derived MEFs from two separate *Ror1^f/f^; Ror2^f/f^; CAG-CreER* embryos and treated each group with either 4-OHT or a vehicle control (Figure 1A). 4-OHT treatment of *Ror1^f/f^; Ror2^f/f^; CAG-CreER* MEFs effectively eliminated Ror1 and Ror2 protein expression, as measured by western blotting, and reduced the phosphorylation of Dvl2, as measured by a motility shift of this protein on SDS-PAGE gels (Figure 1—figure supplement 1). This result confirmed the acute elimination of Ror1 and Ror2 protein expression and a decrease in Wnt5a-Ror-Dvl signaling in both biological replicates analyzed in the MS screen.

**Figure 1—figure supplement 1.**
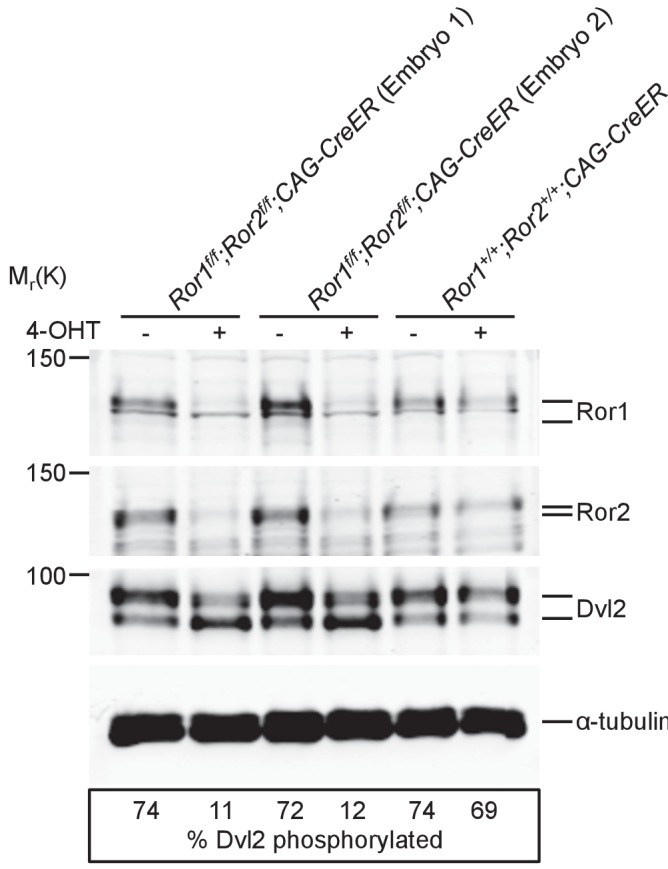
Conditional depletion of Ror1 and Ror2 protein in MEFs. Immunoblots of Ror1 protein, Ror2 protein and Dvl2 protein in primary MEFs derived from the two separate E12.5 *Ror1^f/f^; Ror2^f/f^*; *CAG-CreER* mice and one E12.5 *Ror1^+/+^*; *Ror2^+/+^*; *CAG-CreER* mouse used in the phosphoproteomic screen. Samples were lysed in 8 M urea (see Materials and methods) rather than standard Laemmli sample buffer. 4-OHT or vehicle control was added for 72 hr prior to lysis. α-tubulin was used for loading controls. The asterisk denotes a protein unrelated to Ror1 that cross-reacts with the anti-Ror1 antibody.

**Figure 1—figure supplement 2.**
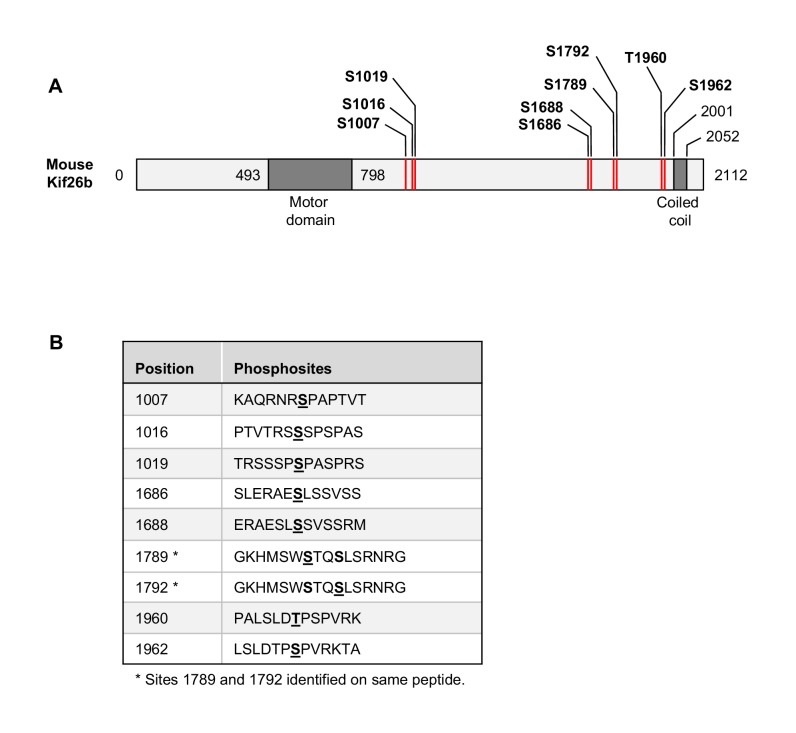
Kif26b phosphorylation sites identified in the phosphoproteomic screen. (**A**) A schematic illustration of mouse Kif26b showing the amino acid boundaries of the motor and coiled coil domains. The motor and coiled coil domains were identified using the following web resources: http://scansite3.mit.edu/; http://www.ch.embnet.org/cgi-bin/COILS_form_parser. The relative positions of the Kif26b phosphorylation sites are marked with the red lines. (**B**) List of the Kif26b phosphorylation sites identified in the phosphoproteomic screen. The phosphorylated amino acids are underlined in bold.

For the last two of the six experimental conditions in the screen, we cultured control MEFs from a single *Ror1^+/+^; Ror2^+/+^; CAG-CreER* embryo and treated these cells with either 4-OHT or a vehicle control to identify any nonspecific effects due to the addition of 4-OHT and the induction of Cre recombinase expression (Figure 1A). As expected, treatment of these control cells with 4-OHT did not alter the expression of Ror proteins or the phosphorylation of Dvl2, as compared with the vehicle control (Figure 1—figure supplement 1). Together, the two experimental replicates and the control condition allowed us to identify changes in the abundance of phosphorylated proteins and/or specific changes in protein phosphorylation events that are due to the disruption of Ror expression.

6498 unique phosphopeptides, representing 7426 distinct phosphosites, were quantified in the screen (Supplementary file 1). For high confidence identification of biochemical changes that are specific to the cells in which the Ror proteins were inducibly knocked out, phosphopeptides categorized as ‘hits’ had to meet the following criteria: (1) an average of ≥2-fold increase or decrease in the abundance of the phosphopeptide in 4-OHT-treated *Ror1^f/f^; Ror2^f/f^; CAG-CreER* MEFs relative to vehicle-treated *Ror1^f/f^; Ror2^f/f^; CAG-CreER* MEFs; (2) a significant fold change (*p*<0.05) across two experimental replicates; and (3) a <2-fold increase or decrease in the abundance of the phosphopeptide in 4-OHT-treated *Ror1^+/+^; Ror2^+/+^; CAG-CreER* MEFs relative to vehicle-treated *Ror1^+/+^; Ror2^+/+^; CAG-CreER* MEFs. The 2-fold threshold was chosen to capture candidates whose change in abundance ranked in the top 0.2 percent of all phosphopeptides analyzed in the screen.

Hits in this screen could reflect two possibilities: (1) Ror signaling mediates the phosphorylation or dephosphorylation of a candidate protein, or (2) Ror signaling alters the total level of expression of a candidate protein. The first possibility is more likely when a specific phosphorylated peptide changes in abundance in a Ror-dependent manner while other phosphopeptides from the same protein do not change. The second possibility is more likely when each phosphorylated peptide from a given protein increases or decreases in abundance in a Ror-dependent manner.

A total of fifteen unique phosphopeptides were identified as hits (Figure 1B and Supplementary file 2). Eleven phosphopeptides increased in abundance and four phosphopeptides decreased in abundance upon Ror depletion. Eight of the phosphopeptides that increased in abundance mapped to the same protein—the kinesin superfamily member Kif26b—making it a high-confidence candidate target of Wnt5a-Ror signaling (Figure 1B,C and Figure 1—figure supplement 2). Moreover, all eight Kif26b phosphopeptides exhibited a significant increase in abundance following genetic ablation of Ror expression in both experimental replicates (Figure 1B and Supplementary file 2). Taken together, these observations strongly suggest that Wnt5a-Ror signaling leads to a decrease in the level of Kif26b protein expression.

### Kif26b is a downstream target of noncanonical Wnt5a-Ror signaling

Kif26b is a highly conserved atypical kinesin of the Kinesin-11 family, which includes Kif26a and Kif26b, two proteins whose developmental and cellular functions have only begun to be revealed in recent years (Uchiyama et al., 2010; Hirokawa and Tanaka, 2015; Guillabert-Gourgues et al., 2016). To further test the hypothesis that Wnt5a-Ror signaling leads to a decrease in Kif26b protein expression, as suggested by our MS screen, we generated polyclonal antibodies that specifically recognize the Kif26b protein. We validated the specificity of these antibodies by western blotting of protein extracts obtained from wild-type MEFs, multiple *Kif26b* shRNA-knockdown MEFs or *Kif26b^-/-^* MEFs (Figure 2—figure supplement 1A–C). We found that the anti-Kif26b antibodies recognized protein bands at the predicted size of Kif26b (~220 kD) in wild-type MEF lysates but not in the *Kif26b* knockout or knockdown MEF lysates, confirming that our antibodies specifically recognize endogenous Kif26b.

**Figure 2.**
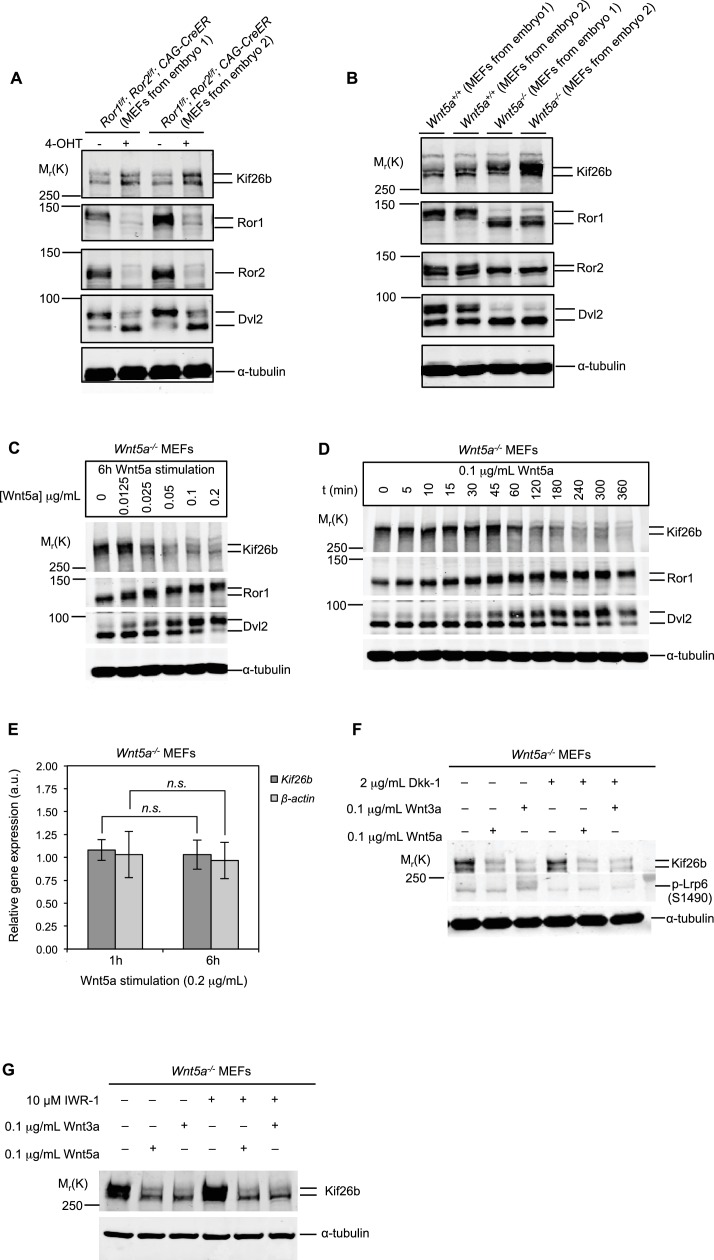
Kif26b protein levels are regulated by the expression of Wnt5a and Ror proteins in primary mouse embryonic fibroblasts. (**A**) Immunoblots of Kif26b protein, Ror1 protein, Ror2 protein and Dvl2 protein in primary MEFs derived from two E12.5 *Ror1^f/f^; Ror2^f/f^*; *CAG-CreER* mice. 4-OHT or vehicle control was added for 72 hr prior to lysis. Results of two independent biological replicates are shown. (**B**) Immunoblots of Kif26b protein, Ror1 protein, Ror2 protein and Dvl2 protein in primary MEFs derived from littermate E12.5 *Wnt5a^+/+^* and *Wnt5a^-/-^* mice. Results of two independent biological replicates are shown. (**C**) Immunoblots of Kif26b protein, Ror1 protein and Dvl2 protein in primary MEFs derived from E12.5 *Wnt5a^-/-^* mice. Recombinant Wnt5a protein was added for 6 hr prior to lysis at the indicated dose. (**D**) Immunoblots of Kif26b protein, Ror1 protein and Dvl2 protein in primary MEFs derived from E12.5 *Wnt5a^-/-^* mice. 0.1 μg/ml recombinant Wnt5a protein was added for the indicated amount of time prior to lysis. (**E**) Plot showing relative mRNA expression of *Kif26b* and *β-actin* as measured by RT-qPCR in primary MEFs derived from E12.5 *Wnt5a^-/-^* mice, with 1 hr or 6 hr of Wnt5a stimulation. The y-axis represents fold change relative to expression levels in unstimulated cells. Error bars represent ± SEM calculated from three technical replicates. t-test (unpaired) was determined for the following comparisons: *Kif26b* 1 hr vs. 6 hr, *p*=0.249, not significant; *β-actin* 1 hr vs. 6 hr, *p*=0.320, not significant. (**F**) Immunoblots of Kif26b protein and phospho-Lrp6 (serine 1490) protein in primary MEFs derived from E12.5 *Wnt5a^-/-^* mice. Recombinant Dkk-1 (0.1 μg/ml) or vehicle control was added 8 hr prior to lysis. Recombinant Wnt3a protein, Wnt5a protein or vehicle control was added for 6 hr prior to lysis. (**G**) Immunoblots of Kif26b protein in primary MEFs derived from E12.5 *Wnt5a^-/-^* mice. IWR-1 or vehicle control was added 7 hr prior to lysis. Recombinant Wnt3a protein, Wnt5a protein or vehicle control was added for 6 hr prior to lysis. α-tubulin was used for loading controls in all experiments. All immunoblot samples were normalized by BCA assays for total protein. 10.7554/eLife.26509.007Figure 2—source data 1.(Related to panel E) Relative mRNA expression of *Kif26b* and *β-actin* as measured by RT-qPCR in primary MEFs derived from E12.5 *Wnt5a*^*-/-*^ mice, after 1 hr or 6 hr of Wnt5a stimulation.

Using these antibodies, we assessed the expression of Kif26b protein in primary MEFs in which Ror1 and Ror2 proteins had been inducibly knocked out. We found that Kif26b levels were elevated in *Ror1^f/f^; Ror2^f/f^; CAG-CreER* MEFs treated with 4-OHT as compared to MEFs with the same genotype treated with a vehicle control (Figure 2A). This finding suggests that Wnt5a-Ror signaling negatively regulates total Kif26b protein expression, as opposed to selectively catalyzing the dephosphorylation of multiple phosphorylation sites across the Kif26b protein. Moreover, this finding validates that our MS screening approach reliably identifies cellular proteins whose expression is regulated by Wnt5a-Ror signaling.

**Figure 2—figure supplement 1.**
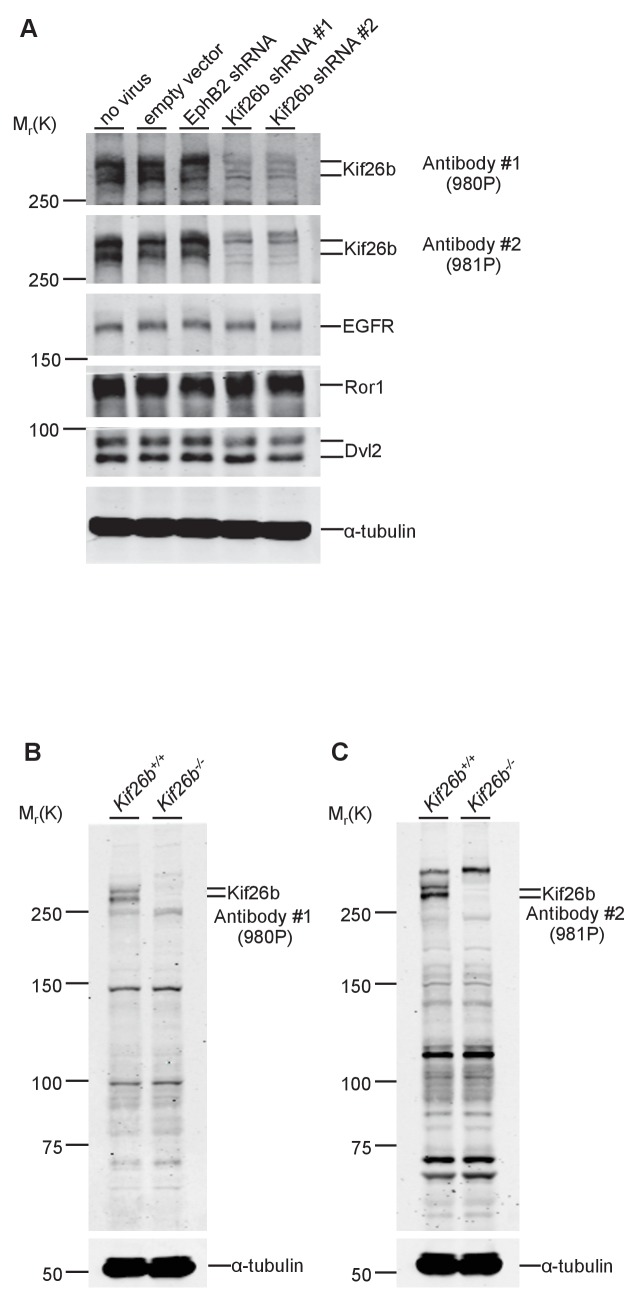
Validation of anti-Kif26b antibodies. (**A**) Immunoblots of Kif26b protein, EGFR protein, Ror1 protein and Dvl2 protein in primary MEFs derived from E12.5 WT mice. Indicated shRNAs were transduced via lentiviral transduction. An empty vector and an shRNA directed at *EphB2* were used as negative controls. (**B**) Immunoblot of Kif26b protein in primary MEFs derived from littermate E12.5 *Kif26b^+/+^* and *Kif26b^-/-^* mice using the anti-Kif26b antibody 980P. (**C**) Immunoblot of Kif26b protein in primary MEFs derived from littermate E12.5 *Kif26b^+/+^* and *Kif26b^-/-^* mice using the anti-Kif26b antibody 981P. α-tubulin was used for loading controls in all experiments.

To test directly whether Wnt5a signaling regulates Kif26b protein levels, we assessed the expression of Kif26b in primary MEFs in which *Wnt5a* had been knocked out. We observed that *Wnt5a^-/-^* MEFs also had a higher level of Kif26b protein relative to wild-type control MEFs, indicating that Wnt5a, like Ror1 and Ror2, negatively regulates the steady-state level of Kif26b expression (Figure 2B). This finding suggests that Wnt5a signaling via Ror proteins leads to a decrease in Kif26b protein expression.

We next investigated whether acute activation of Wnt5a-Ror signaling by the addition of exogenous Wnt5a triggers a decrease in Kif26b protein expression in MEFs. We stimulated *Wnt5a^-/-^* MEFs with purified, recombinant Wnt5a and found that this treatment led to a decrease in Kif26b protein expression in a dose-dependent manner (Figure 2C). The decrease in Kif26b was accompanied by a commensurate increase in Ror1 and Dvl2 phosphorylation, a readout of Wnt5a-Ror signaling (Figure 2C). These findings suggest that Wnt5a induces the downregulation of endogenous Kif26b expression as the Wnt5a-Ror-Dvl pathway becomes activated.

We found that the Wnt5a-induced downregulation of Kif26b expression is first detected 1 hr after Wnt5a stimulation and that Kif26b expression is maximally decreased after 6 hr (Figure 2D). The kinetics of Kif26b protein downregulation closely paralleled those of Ror1 and Dvl2 phosphorylation in response to acute Wnt5a stimulation (Figure 2D), suggesting that Wnt5a signaling regulates these downstream biochemical events in a coordinated manner. In addition, we found that stimulation with Wnt5a does not change the level of *Kif26b* mRNA in *Wnt5a^-/-^* MEFs, as assayed by reverse transcription-quantitative PCR (RT-qPCR), throughout the course of the experiment (Figure 2E), suggesting that Wnt5a-dependent downregulation of Kif26b protein levels occurs post-transcriptionally. Together, these findings establish that Wnt5a-Ror signaling leads to a decrease in the steady-state levels of Kif26b protein expression in MEFs and validate Kif26b as a *bona fide* target of the Wnt5a-Ror signaling pathway.

The Wnt5a-Ror pathway is generally thought to operate via a noncanonical, β-catenin-independent mechanism (Green et al., 2008b). To determine whether Wnt5a-Ror signaling induces the downregulation of Kif26b expression via a noncanonical Wnt signaling mechanism, we tested whether blocking the canonical Wnt/β-catenin pathway with Dkk-1, an antagonist of the β-catenin-dependent Wnt pathway that binds and prevents the phosphorylation of the canonical Wnt signaling pathway co-receptors Lrp5 and Lrp6, inhibits the Wnt5a-induced decrease in Kif26b protein in MEFs (Bafico et al., 2001). We found that exposure of *Wnt5a^-/-^* MEFs to exogenous Wnt5a protein induced Kif26b downregulation to a similar degree with and without the addition of Dkk-1 (Figure 2F). To ensure that the Dkk-1 protein used in the experiment was active, we assessed whether the same concentration of Dkk-1 was capable of blocking signaling by Wnt3a, a prototypic canonical Wnt that induces the phosphorylation of the Wnt receptor Lrp6. We found that Wnt3a-dependent phosphorylation of Lrp6 was completely blocked in the presence of Dkk-1 (Figure 2F). These findings suggest that Wnt5a-dependent regulation of Kif26b occurs via a noncanonical Wnt signaling mechanism that is independent of the canonical Wnt/β-catenin pathway.

To verify that Wnt5a regulation of Kif26b occurs via a noncanonical Wnt signaling mechanism, we next tested whether inhibiting the Wnt/β-catenin pathway at a more downstream step blocks Wnt5a-dependent downregulation of Kif26b levels. For this purpose, we used IWR-1, a small molecule that inhibits Wnt/β-catenin signaling by stabilizing Axin2, a key component of the β-catenin destruction complex (Lee et al., 2003; Chen et al., 2009). Similar to our findings using Dkk-1 to block the canonical Wnt/β-catenin pathway at the receptor level, we found that pre-treatment of *Wnt5a^-/-^* MEFs with IWR-1 did not block the ability of Wnt5a or Wnt3a to induce the downregulation of Kif26b protein expression (Figure 2G). These results further support the conclusion that Wnt5a-Ror signaling leads to the downregulation of Kif26b protein expression via a noncanonical, β-catenin-independent mechanism.

It is interesting to note that the addition of exogenous Wnt3a protein, classically considered a canonical Wnt, also led to a decrease in the expression of Kif26b in *Wnt5a^-/-^* MEFs, even in the presence of Dkk-1 or IWR-1 (Figure 2F,G). These findings indicate that exogenous Wnt3a also signals via a noncanonical Wnt signaling mechanism to downregulate the expression of Kif26b and supports the emerging view that the distinction between canonical and noncanonical Wnt signaling is not strictly determined at the level of Wnt ligands (van Amerongen et al., 2008). However, it is also possible that there is some specificity to Wnt3a and Wnt5a signaling in the developing embryo that is lost when these factors are studied using cultured MEFs.

### Wnt5a signals the degradation of Kif26b via the ubiquitin-proteasome pathway

We next investigated the biochemical mechanisms by which Wnt5a-Ror signaling leads to decreased Kif26b protein expression. To more accurately quantify Kif26b levels in live cells, we developed a flow cytometry-based reporter assay using NIH/3T3 cell lines stably expressing a GFP-Kif26b fusion protein. We chose NIH/3T3 cells for the reporter assay because these cells express key components of the Wnt5a-Ror pathway, including Ror1, Ror2, Dvl2 and Kif26b, which we found to be similarly regulated by Wnt5a as in MEFs (Figure 3—figure supplement 1A,B). Furthermore, NIH/3T3 cells are an immortalized cell line derived from mouse embryonic mesenchymal cells that undergo morphogenetic movements during development (Todaro and Green, 1963), and they have been used previously to study cell behaviors in the context of Wnt5a-Ror signaling (Endo et al., 2012). Western analysis and immunostaining of these NIH/3T3 cell lines confirmed that the GFP-Kif26b protein is stably expressed in these cells (Figure 3—figure supplement 2A–C).

**Figure 3.**
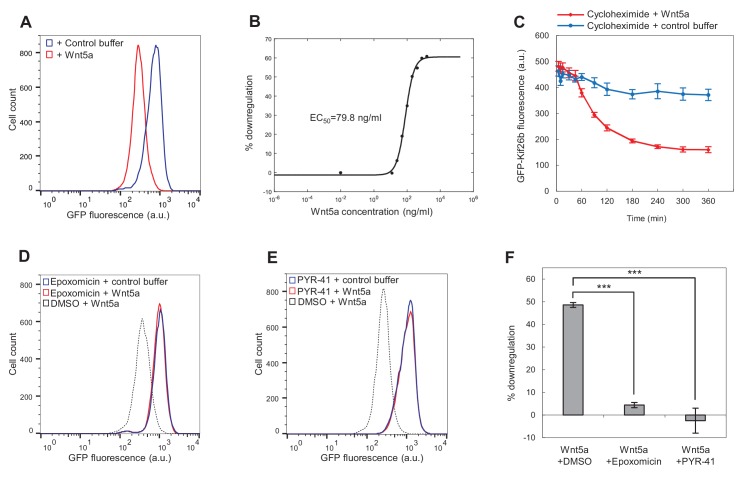
Wnt5a downregulates Kif26b levels via a ubiquitin/proteasome-dependent mechanism. (**A**) Flow cytometry histograms depicting the downregulation of GFP-Kif26b fluorescence in the WRK reporter cell line after Wnt5a stimulation (0.2 μg/ml Wnt5a) for 6 hr. (**B**) Dose-response curve showing GFP-Kif26b downregulation as a function of Wnt5a concentration, as measured in the WRK reporter assay. (**C**) The kinetics of GFP-Kif26b turnover in the absence or presence of Wnt5a stimulation, as measured in the WRK reporter assay. Cycloheximide was used to block new protein synthesis in the reporter cells. (**D**) Flow cytometry histograms depicting the effect of epoxomicin treatment (1 μM) on the ability of Wnt5a (0.2 μg/ml) to downregulate GFP-Kif26b fluorescence in the WRK reporter assay. DMSO was used as the drug vehicle control. (**E**) Flow cytometry histograms depicting the effect of PYR-41 treatment (50 μM) on the ability of Wnt5a (0.2 μg/ml) to downregulate GFP-Kif26b fluorescence in the WRK reporter assay. DMSO was used as the drug vehicle control. (**F**) Quantification of the effects of DMSO, epoxomicin and PYR-41 on the ability of Wnt5a to downreagulate GFP-Kif26b fluorescence in the WRK reporter assay, as shown in (**D**) and (**E**). Error bars represent ± SEM calculated from three technical replicates. t-tests were determined for the following comparisons: DMSO vs. epoxomicin, *p*<0.001; DMSO vs. PYR-41, *p*<0.001. 10.7554/eLife.26509.011Figure 3—source data 1.(Related to panel C) The kinetics of GFP-Kif26b turnover in the absence or presence of Wnt5a stimulation. 10.7554/eLife.26509.012Figure 3—source data 2.(Related to panel F) The effects of DMSO, epoxomicin and PYR-41 on the ability of Wnt5a to downregulate GFP-Kif26b fluorescence in the WRK reporter assay.

Given that Wnt5a signaling acutely downregulates the level of endogenous Kif26b expression in MEFs, we tested whether Wnt5a similarly downregulates GFP-Kif26b levels in the reporter NIH/3T3 cells. Treatment of these cells with exogenous recombinant Wnt5a protein induced an approximately 50% decrease in GFP-Kif26b expression as detected by three independent methods: flow cytometry (Figure 3A), western analysis (Figure 3—figure supplement 2A) and time-lapse microscopy (Video 1). These results indicate that Wnt5a treatment leads to a decrease in GFP-Kif26b in NIH/3T3 cells similar to that observed for endogenous Kif26b in MEFs, and that the GFP-Kif26b reporter can be used reliably to monitor the decrease in Kif26b protein expression in response to Wnt5a signaling. To the best of our knowledge, this is the first fluorescence-based reporter for real-time measurement of a non-transcriptional Wnt5a-Ror signaling response in live cells. For the remainder of this article, the *W*nt5a-*R*or-*K*if26b reporter assay will be referred to as the WRK reporter assay.

**Figure 3—figure supplement 1.**
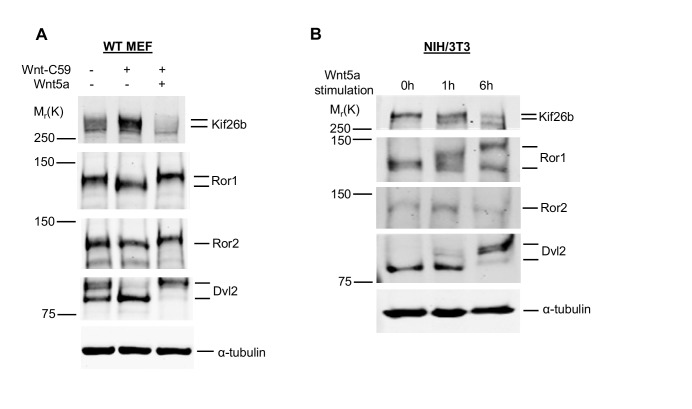
Expression of Wnt5a-Ror signaling components in MEFs and NIH/3T3 cells. (**A**) Immunoblots of Kif26b protein, Ror1 protein, Ror2 protein and Dvl2 protein in primary MEFs derived from E12.5 WT mice. WT MEFs have a high basal level of Wnt5a-Ror signaling activity, as indicated by hyperphosphorylation of Ror1, Ror2 and Dvl2. Wnt-C59 was added to inhibit this basal activity. The effects of Wnt-C59 on Kif26b levels and the phosphorylation of Ror1, Ror2 and Dvl2 can be rescued by treatment with recombinant Wnt5a (0.2 μg/ml Wnt5a for 6 hr). (**B**) Immunoblots of Kif26b protein, Ror1 protein, Ror2 protein and Dvl2 protein in NIH/3T3 cells. NIH/3T3 cells have a low basal level of Wnt5a-Ror signaling. Wnt5a was added to stimulate this activity. α-tubulin was used for loading controls in all experiments.

**Figure 3—figure supplement 2.**
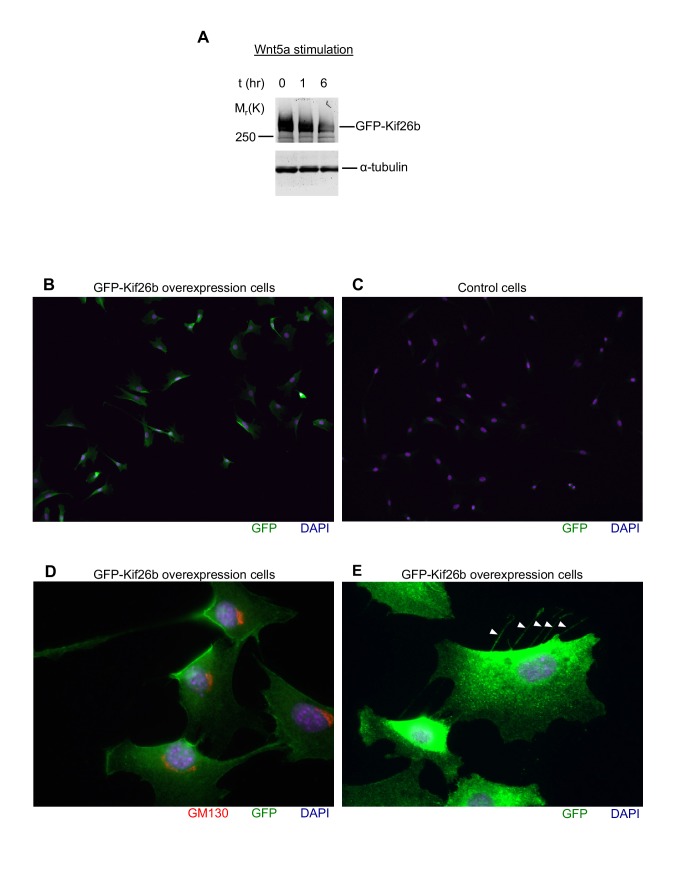
Characterization of the GFP-Kif26b (WRK) reporter line. (**A**) Anti-Kif26b immunoblot of the GFP-Kif26b protein expressed under the control of an EF1 promoter in the WRK reporter cell line. The reporter cells were stimulated with 0.2 μg/ml Wnt5a for 0 hr, 1 hr or 6 hr. α-tubulin was used for loading controls. (**B and C**) Immunostaining of the GFP-Kif26b expressing NIH/3T3 cell line (**B**) and a control NIH/3T3 cell line for GFP (**C**). Images were acquired with a 10x objective at equal exposures. (**D**) Co-immunostaining of a GFP-Kif26b expressing NIH/3T3 cell line for GFP and the *cis* Golgi network marker GM130, which typically localizes to the leading side of the nucleus of polarized mesenchymal cells. Image was acquired with a 63x objective. (**E**) Immunostaining of a GFP-Kif26b expressing NIH/3T3 cell line for GFP. Image was acquired with a 63x objective. Exposure is increased to highlight GFP staining in retraction fibers (arrowheads).

**Video 1.** Time-lapse fluorescent confocal microscopy reveals rapid Wnt5a-dependent downregulation of the GFP-Kif26b signal in the NIH/3T3 reporter cell line. Bright signal reflects GFP-Kif26b fluorescence. Buffer or 0.2 μg/mL recombinant Wnt5a was added at the start of the imaging session. Cells were imaged every 10 min for 16 hr at 40x magnification.

We next used the WRK reporter assay in conjunction with flow cytometry to determine the dose-response relationship between Wnt5a and GFP-Kif26b. We found that nanomolar amounts of Wnt5a induced the downregulation of GFP-Kif26b expression with a calculated EC_50_ of 79.8 ng/ml (or 2.1 nM; Figure 3B). This response to Wnt5a occurs at a Wnt5a concentration that is similar to that of other previously reported Wnt-induced cellular responses (Bryja et al., 2007a, 2007b; Witze et al., 2008; Ho et al., 2012; van Amerongen et al., 2012; Witze et al., 2013; Park et al., 2015; Connacher et al., 2017), suggesting that Wnt5a-induced Kif26b downregulation is a physiologically relevant response to Wnt5a.

Wnt5a-Ror signaling could downregulate the steady state level of Kif26b protein either by decreasing Kif26b synthesis or by increasing Kif26b turnover. Given that exposure of fibroblasts to Wnt5a leads to a decrease in Kif26b expression over minutes to hours but does not lead to a reduction in the level of *Kif26b* mRNA (Figure 2D,E), we favored the latter possibility. To directly measure the rate of Kif26b turnover, we treated WRK reporter cells with cycloheximide to block new protein synthesis and then used flow cytometry to measure the effect of Wnt5a treatment on the rate of GFP-Kif26b turnover. Consistent with the hypothesis that Wnt5a treatment leads to the increased degradation of Kif26b, we found that Wnt5a accelerated the turnover of GFP-Kif26b in the reporter cells (Figure 3C). In the absence of Wnt5a stimulation, 80.2% of the GFP-Kif26b signal remained in the cells 6 hr after the initiation of the cycloheximide treatment. By contrast, in the presence of Wnt5a stimulation, only 33.2% of the GFP-Kif26b signal remained in the same period. These results strongly suggest that Wnt5a treatment leads to a decrease in the steady-state levels of Kif26b expression in cells by promoting Kif26b turnover.

We next investigated whether Wnt5a downregulates Kif26b expression by increasing the rate of Kif26b degradation via the ubiquitin-proteasome system (UPS). To test this hypothesis, we asked whether a selective inhibitor of the proteasome, epoxomicin, blocks downregulation of Kif26b expression in response to Wnt5a (Meng et al., 1999). Pre-treatment of the WRK reporter line with epoxomicin strongly blocked Wnt5a-induced Kif26b downregulation (Figure 3D,F). This result suggests that Wnt5a-induced downregulation of Kif26b occurs via proteasome-dependent degradation.

To determine whether protein ubiquitination is specifically required for Wnt5a-induced Kif26b degradation, we tested whether an inhibitor of the E1 enzyme required for ubiquitin activation, PYR-41, also blocks Wnt5a-induced Kif26b downregulation (Yang et al., 2007). Pre-treatment of the WRK reporter cells with PYR-41 blocked Wnt5a-induced Kif26b downregulation to an extent similar to that observed upon epoxomicin treatment (Figure 3E,F). This finding provides further evidence that downregulation of Kif26b expression induced by Wnt5a occurs via the UPS. We conclude from these results that exposure to Wnt5a leads to the downregulation of the steady-state levels of Kif26b expression by promoting Kif26b ubiquitin- and proteasome-dependent degradation.

### Frizzled and Dishevelled proteins mediate Wnt5a-induced Kif26b degradation

Our pharmacological inhibitor experiments suggest that the WRK reporter assay might be used to interrogate other molecular components of the Wnt5a-Ror signaling pathway that operate upstream of Kif26b. To test this idea, we first investigated a possible role of Frizzled (Fzd) proteins in Wnt5a-dependent Kif26b degradation. Fzds make up a family of 10 seven-transmembrane domain receptor proteins that function as co-receptors in canonical Wnt/β-catenin signaling and as polarity determinants in the PCP pathway (Vinson and Adler, 1987; Bhanot et al., 1996). Recent work additionally implicates Fzd proteins in aspects of noncanonical Wnt function as well as in the phosphorylation of Ror2 in cultured cells (Habas et al., 2001; Nishita et al., 2010; Grumolato et al., 2010; Sato et al., 2010). Moreover, protein sequence homology analysis and in vivo mouse genetic studies revealed that Fzd1, Fzd2 and Fzd7 form a distinct sub-family that functions redundantly to control tissue morphogenetic events such as convergent extension of embryonic tissues and closure of the palate and ventricular septum (Yu et al., 2010; Yu et al., 2012). As many of these developmental processes also require the Wnt5a-Ror pathway, we hypothesized that certain Fzd proteins, such as those in the Fzd1, Fzd2 and Fzd7 sub-family, might participate in Wnt5a-Ror-Kif26b signaling.

**Figure 4.**
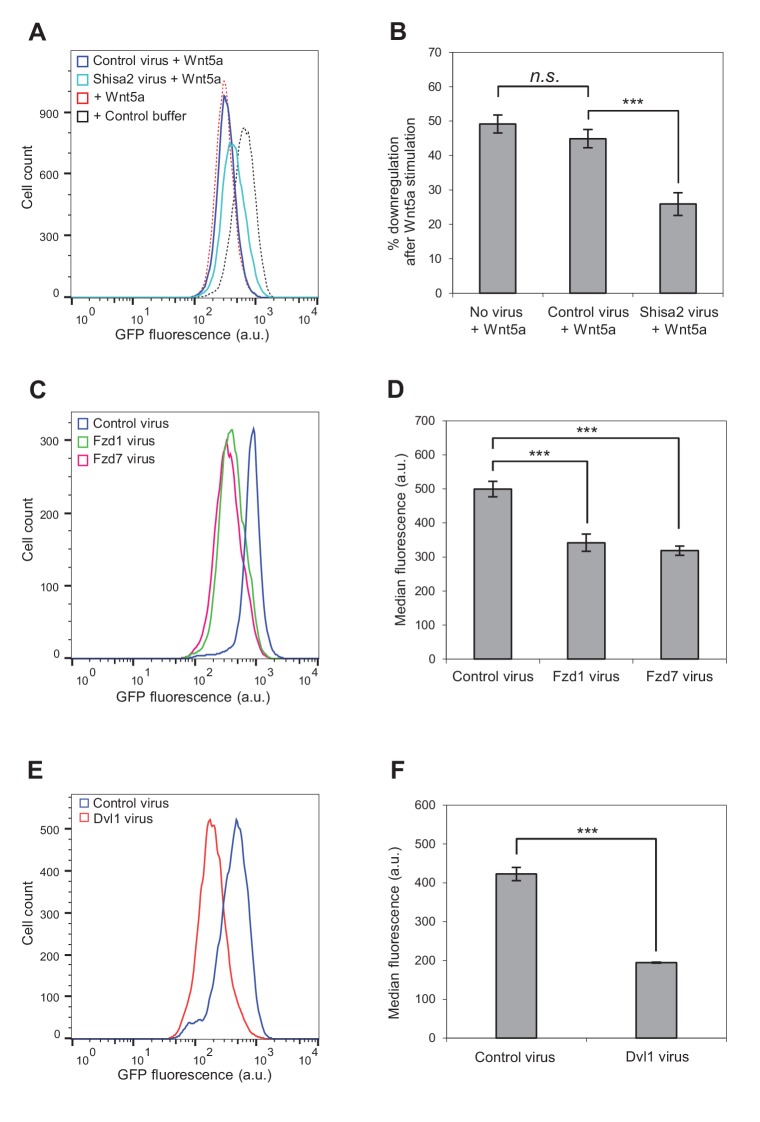
Involvement of Fzd and Dvl proteins in Kif26b degradation. (**A**) Flow cytometry histograms depicting the effect of ectopic Shisa2 expression on the ability of Wnt5a to induce GFP-Kif26b degradation in the WRK reporter assay. Mouse Shisa2 was expressed via lentiviral transduction. A lentivirus expressing Cas9 was used as the control. The effect of Wnt5a or control buffer treatment on the WRK reporter line without virus infection is shown as a reference. (**B**) Quantification of effects of the control virus (Cas9) and the Shisa2 virus on the ability of Wnt5a to downregulate the GFP-Kif26b fluorescence in the WRK reporter assay, as shown in (**A**). Error bars represent ± SEM calculated from three technical replicates. t-tests were determined for the following comparisons: control virus vs. Shisa2 virus, *p*<0.001; control virus vs. no virus, *p*=0.0957 (not significant). (**C**) Flow cytometry histograms depicting the effect of ectopic Fzd1 or Fzd7 expression on GFP-Kif26b levels in the WRK reporter assay. Mouse Fzd1 and Fzd7 were expressed via lentiviral transduction. A lentivirus expressing Cas9 was used as the control. (**D**) Quantification of the median GFP-Kif26b fluorescence in the WRK reporter cell line infected with a Fzd1 virus, a Fzd7 virus, or a Cas9 control virus. Error bars represent ± SEM calculated from six technical replicates. t-tests were determined for the following comparisons: control virus vs. Fzd1 virus, *p*<0.001; control virus vs. Fzd7 virus, *p*<0.001. (**E**) Flow cytometry histograms depicting the effect of ectopic Dvl1 expression on GFP-Kif26b levels in the WRK reporter assay. Dvl1 was expressed via lentiviral transduction. A lentivirus expressing Cas9 was used as the control. (**F**) Quantification of the median GFP-Kif26b fluorescence in the WRK reporter cell line infected with a Dvl1-expressing virus, or a Cas9-expressing control virus. Error bars represent ± SEM calculated from three technical replicates. t-test was determined for control virus vs. Dvl1 virus, *p*<0.001. 10.7554/eLife.26509.016Figure 4—source data 1.(Related to panel B) The effects of the control virus and the Shisa2 virus on the ability of Wnt5a to downregulate GFP-Kif26b fluorescence in the WRK reporter assay. 10.7554/eLife.26509.017Figure 4—source data 2.(Related to panel D) The median GFP-Kif26b fluorescence in the WRK reporter cell line infected with a Fzd1 virus, a Fzd7 virus, or a Cas9 control virus. 10.7554/eLife.26509.018Figure 4—source data 3.(Related to panel F) Quantification of the median GFP-Kif26b fluorescence in the WRK reporter cell line infected with a Dvl1 virus or a Cas9 control virus.

To test whether Fzd proteins mediate Wnt5a-Ror-Kif26b signaling, we first took a loss-of-function approach using Shisa proteins, which are Wnt signaling regulators that inhibit Fzd processing and trafficking by sequestering Fzds in the endoplasmic reticulum (Yamamoto et al., 2005). We found that viral transduction of Shisa2 expression, but not Cas9 expression as a control, partially blocked the ability of Wnt5a to induce Kif26b degradation in the WRK reporter cell line (Figure 4A,B). This observation suggests that the Fzd family plays a role in Wnt5a-dependent regulation of Kif26b.

**Figure 4—figure supplement 1.**
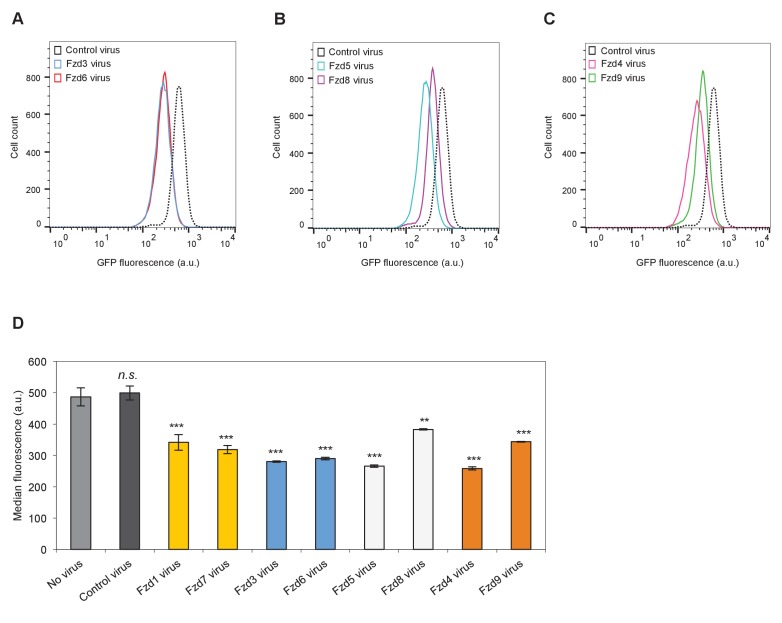
Comparison of the abilities of distinct Fzd subfamily members to induce Kif26b degradation. (**A**) Flow cytometry histograms depicting the effect of ectopic Fzd3 or Fzd6 expression on GFP-Kif26b levels in the WRK reporter assay. Mouse Fzd3 and Fzd6 were expressed via lentiviral transduction. Histogram from an uninfected WRK reporter cell line is shown as a reference. (**B**) Flow cytometry histograms depicting the effect of ectopic Fzd5 or Fzd8 expression on GFP-Kif26b levels in the WRK reporter assay. Mouse Fzd5 and Fzd8 were expressed via lentiviral transduction. Histogram from an uninfected WRK reporter cell line is shown as a reference. (**C**) Flow cytometry histograms depicting the effect of ectopic Fzd4 or Fzd9 expression on GFP-Kif26b levels in the WRK reporter assay. Mouse Fzd4 and Fzd9 were expressed via lentiviral transduction. Histogram from an uninfected WRK reporter cell line is shown as a reference. (**D**) Quantification of the median GFP-Kif26b fluorescence in the WRK reporter cell line infected with various Fzd viruses shown in (**A**) – (**C**). Fzds with the same color bars are part of the same sub-families. WRK reporter lines uninfected or infected with a Cas9 virus are shown as controls. Error bars represent ± SEM calculated from technical replicates. n = 9 for no virus and control viurs. n = 6 for Fzd1 and Fzd7 viruses. n = 3 for all other Fzd viruses. t-tests were determined for the following comparisons: control virus vs. Fzd1 virus, *p*<0.001; control virus vs. Fzd7 virus, *p*<0.001; control virus vs. Fzd3 virus, *p*<0.001; control virus vs. Fzd6 virus, *p*<0.001; control virus vs. Fzd5 virus, *p*<0.001; control virus vs. Fzd8 virus, *p*<0.01; control virus vs. Fzd4 virus, *p*<0.001; control virus vs. Fzd9 virus, *p*<0.001; control virus vs. no virus, *p*=0.3683 (not significant).

To more directly test the involvement of Fzds in Wnt5a-Kif26b signaling, we asked whether overexpression of Fzds could induce Kif26b degradation in the absence of exogenously added Wnt5a. We first focused on members of the Fzd1, Fzd2 and Fzd7 sub-family, as the mouse knockout phenotypes of these Fzds are most consistent with a functional interaction with the Wnt5a-Ror pathway (Yu et al., 2010; Yu et al., 2012). Interestingly, we found that lentiviral transduction of Fzd1 or Fzd7 expression, but not of the negative control Cas9, constitutively induced Kif26b degradation as measured in the WRK assay (Figure 4C,D). This finding, taken together with the decreased Wnt5a-Ror-Kif26b signaling upon expression of the Fzd inhibitor Shisa2, strongly suggests that Fzd family proteins are involved in Wnt5a regulation of Kif26b degradation, possibly as co-receptors together with members of the Ror family of proteins.

We also considered the possibility that functional specificity might exist among the different Fzd sub-families. We therefore used the WRK assay to test multiple members from each Fzd sub-family, as defined previously (Yu et al., 2010; Yu et al., 2012). Interestingly, we found that all the Fzd family members that we tested were able to induce Kif26b degradation (Figure 4—figure supplement 1). However, it is possible that the specificity of Fzd protein function is lost when these proteins are overexpressed.

We next investigated the role of Dvl proteins in Wnt5a-Ror-dependent Kif26b degradation. Our previous study identified Dvl phosphorylation as a specific downstream target of Wnt5a-Ror signaling (Ho et al., 2012), and a recent study demonstrated that the Dvl and Kif26b proteins physically interact (Guillabert-Gourgues et al., 2016). Moreover, we found that Wnt5a-induced Kif26b degradation occurs with similar kinetics as Wnt5a-induced Dvl phosphorylation (Figure 2D). However, it remains unknown whether Dvl proteins are required for Wnt5a-dependent degradation of Kif26b. Since the presence of three *Dvl* genes in the mammalian genome makes loss-of-function analysis of Dvl proteins challenging, we took a gain-of-function approach to determine if Dvl protein expression affects Wnt5a-Ror-dependent Kif26b degradation. Notably, a similar overexpression approach was previously used to demonstrate a role for Dvl proteins in Wnt/β-catenin signaling, as overexpression of Dvl proteins in *Xenopus* embryos induces axis duplication recapitulating overexpression of canonical Wnts (Sokol et al., 1995). Using the WRK reporter assay, we found that overexpression of Dvl1, but not overexpression of a control protein Cas9, led to an increase in Kif26b degradation (Figure 4E,F), mimicking the effects of Wnt5a stimulation or Fzd overexpression. Taken together, these findings establish a functional role for both Fzd receptors and Dvl1 (and likely other Dvl proteins) in Wnt5a-Ror-dependent Kif26b degradation.

### The Wnt5a-Ror-Kif26b signaling cassette directs the migratory behavior of cells

Genetic studies in *C. elegans* have shown that mutations in the nematode orthologs of *Kif26b* (*vab-8*) and *Ror* (*cam-1*) cause similar polarized cell migration and axon guidance phenotypes, and a recent study in human umbilical vein endothelial cells (HUVECs) demonstrates a physical interaction between Kif26b and Dvl3 (Forrester et al., 1998; Wolf et al., 1998; Chien et al., 2015; Guillabert-Gourgues et al., 2016). These findings, together with the observations reported above, raise the possibility that the cell biological effects of noncanonical Wnt signaling are mediated by the Wnt5a-Ror-dependent degradation of Kif26b. To test this hypothesis, we employed both gain- and loss-of-function approaches in cultured NIH/3T3 cells, the same cells used for the WRK assays. Western analysis of the NIH/3T3 cell lines used in the WRK assay showed that GFP-tagged Kif26b protein is overexpressed relative to endogenous Kif26b in NIH/3T3 cells (Figure 5—figure supplement 1A), indicating that these cells could be used to assess the effects of Kif26b overexpression on cell responses. For examining the effects of loss of Kif26b expression on cellular responses in NIH/3T3 cells, we used CRISPR/Cas9-mediated genome editing to generate stable cell lines in which the expression of *Kif26b* is knocked out (Figure 5—figure supplement 1B and C). Western blot analysis verified complete elimination of Kif26b protein relative to a control cell line expressing the Cas9 endonuclease without a guide RNA (Figure 5—figure supplement 1B). We next confirmed that neither GFP-Kif26b overexpression nor loss of Kif26b affects cell proliferation or survival through mitotic index quantification and TUNEL staining, respectively (Figure 5—figure supplement 1D–G), indicating that these NIH/3T3 cell lines could be used to study other possible Kif26b-dependent cellular responses.

**Figure 5.**
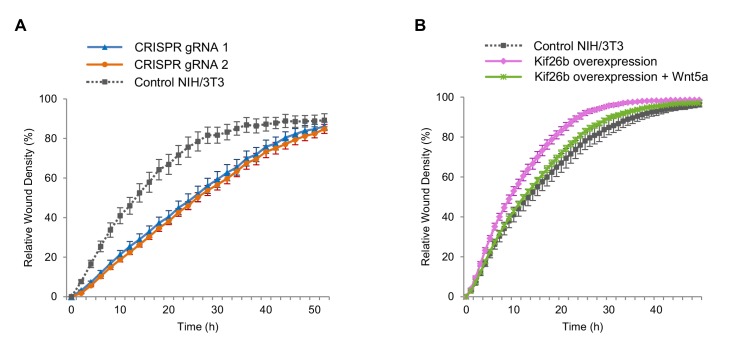
Wnt5a-Kif26b signaling modulates the migratory behavior of NIH/3T3 cells. (**A**) Relative wound density of two separate NIH/3T3 cell lines in which Kif26b expression is knocked out using CRISPR/Cas9 genome editing, and one control Cas9-expressing NIH/3T3 cell line in a kinetic wound-healing assay. Data are the mean of five independent samples and error bars indicate SEM. t-tests (unpaired) were determined for the following comparisons: control NIH/3T3 vs. gRNA1, *p*<0.05; control NIH/3T3 vs. gRNA2, *p*<0.05. (**B**) Relative wound density of a GFP-Kif26b expressing NIH/3T3 cell line, treated with or without Wnt5a (0.2 μg/ml, added at the 0 hr time point) and a control NIH/3T3 cell line in a kinetic wound-healing assay. Data are the mean of five independent samples and error bars indicate SEM. t-tests (unpaired) were determined for the following comparisons: control NIH/3T3 vs. Kif26b overexpression, *p*<0.05 (at every time point except the last time point); Kif26b overexpression vs. Kif26b overexpression +Wnt5a, *p*<0.05 (from 3 to 38 hr time points). 10.7554/eLife.26509.023Figure 5—source data 1.(Related to panel A) Relative wound density of two separate NIH/3T3 cell lines in which Kif26b expression is knocked out using CRISPR/Cas9 genome editing, and one control Cas9-expressing NIH/3T3 cell line in a kinetic wound-healing assay. 10.7554/eLife.26509.024Figure 5—source data 2.(Related to panel B) Relative wound density of a GFP-Kif26b expressing NIH/3T3 cell line, treated with or without Wnt5a and a control NIH/3T3 cell line in a kinetic wound-healing assay.

We employed an automated kinetic wound-healing assay that provides a quantitative, integrated readout of cell morphogenesis such as cell polarization, cell motility and cell adhesion by measuring the wound closure efficiency of cells under different experimental conditions (Gujral et al., 2014a). The wound-healing assay has been used previously to assess effects of noncanonical Wnt signaling (Gujral et al., 2014a, 2014b). Using this assay, we asked if the level of Kif26b expression affects wound closure efficiency. We found that Kif26b-knockout cells exhibit a decrease in wound closure efficiency relative to control cells while cell lines overexpressing GFP-Kif26b exhibit an enhanced rate of wound closure efficiency relative to control cells (Figure 5A,B). These findings suggest that Kif26b may promote cell migration and are consistent with previous observations in HUVECs where Kif26b expression was correlated with increased directional cell migration (Guillabert-Gourgues et al., 2016). Taken together, these findings suggest that by controlling the rate of Kif26b protein degradation, Wnt5a-Ror signaling regulates cell migration.

**Figure 5—figure supplement 1.**
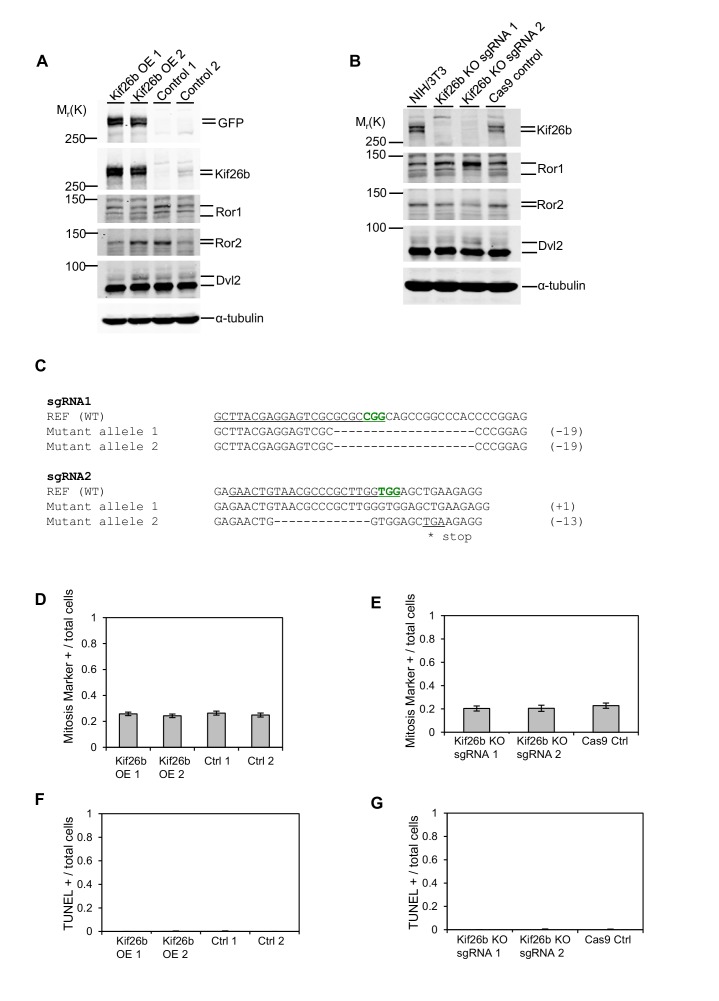
Overexpression of GFP-Kif26b or knockout of Kif26b expression does not affect the proliferation rate or survival of NIH/3T3 cells. (**A**) Immunoblots of Kif26b protein, Ror1 protein, Ror2 protein and Dvl2 protein in NIH/3T3 cell lines overexpressing (OE) GFP-tagged Kif26b protein using the Flp-In system or control NIH/3T3 cell lines that were transfected with an empty vector and selected in parallel. (**B**) Immunoblots of Kif26b protein, Ror1 protein, Ror2 protein and Dvl2 protein in NIH/3T3 cells, two separate NIH/3T3 cell lines in which Kif26b expression is knocked out using CRISPR/Cas9 genome editing, and one control Cas9-expressing NIH/3T3 cell line. (**C**) Sequences of the CRISPR/Cas9-generated mutated Kif26b alleles. The PAM site for each target sequence is marked in green. (**D**) Fraction of cells exhibiting nuclear staining of phosphorylated Serine 10 of Histone H3 relative to total DAPI-positive nuclei of NIH/3T3 cell lines expressing GFP-tagged Kif26b protein and control NIH/3T3 cell lines. (**E**) Fraction of cells exhibiting nuclear staining of phosphorylated Serine 10 of Histone H3 relative to total DAPI-positive nuclei of NIH/3T3 cell lines in which Kif26b expression is knocked out using CRISPR/Cas9 genome editing and a control Cas9-expressing. NIH/3T3 cell line. (**F**) Fraction of cells exhibiting nuclear TUNEL staining relative to total DAPI-positive nuclei of NIH/3T3 cell lines expressing GFP-tagged Kif26b protein and control NIH/3T3 cell lines. (**G**) Fraction of cells exhibiting nuclear TUNEL staining relative to total DAPI-positive nuclei of NIH/3T3 cell lines in which Kif26b expression is knocked out using CRISPR/Cas9 genome editing and a control Cas9-expressing NIH/3T3 cell line. Error bars represent ± SEM for each condition.

**Figure 5—figure supplement 2.**
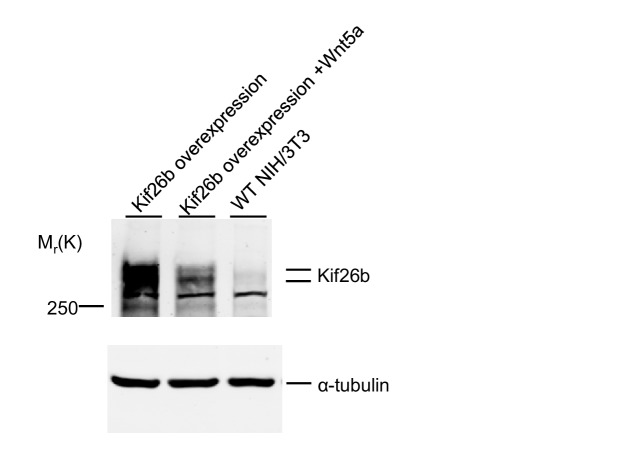
Wnt5a downregulates the cellular levels of GFP-Kif26b. Immunoblots showing levels of Kif26b in GFP-Kif26b-overexpressing NIH/3T3 cells, without or with Wnt5a stimulation (0.1 μg/ml Wnt5a for 24 hr). The level of Kif26b endogenously expressed in WT NIH/3T3 cells is also shown. α-tubulin was used as a protein loading control.

**Figure 5—figure supplement 3.**
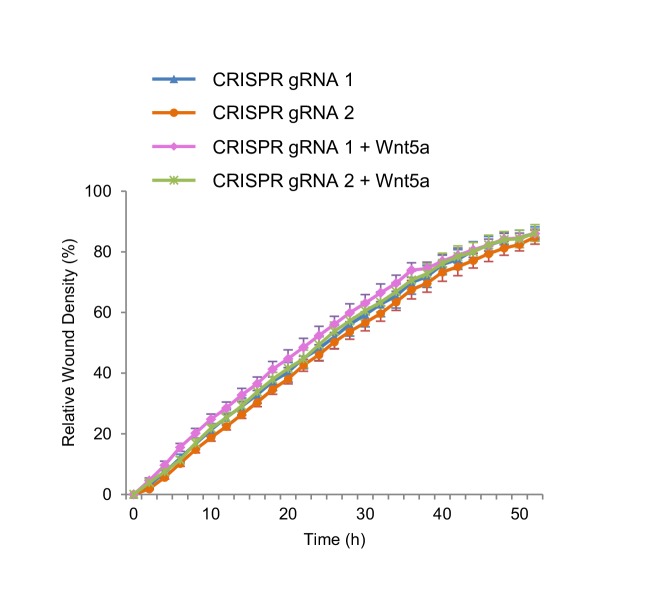
Wnt5a treatment has no effect on the wound closure efficiency of Kif26b knockout NIH/3T3 cells. Relative wound density of two separate NIH/3T3 cell lines in which Kif26b expression is knocked out using CRISPR/Cas9 genome editing, without or with Wnt5a treatment. Traces of Kif26b knockout cell lines without Wnt5a treatment shown in this figure are the same traces as shown in Figure 5A.

Given that exposure of NIH/3T3 cells to Wnt5a acutely downregulates Kif26b protein expression, we next tested whether this treatment affects the migration of these cells in the wound-healing assay. We found that Wnt5a treatment decreases the wound closure efficiency of cells overexpressing GFP-Kif26b to a rate approximating that of a control NIH/3T3 cell line in which GFP-Kif26b is not overexpressed (Figure 5B). This Wnt5a-mediated decrease in wound closure efficiency was correlated with a concomitant decrease in the cellular abundance of Kif26b, suggesting that the specific degradation of Kif26b could underlie the decrease in wound closure efficiency (Figure 5—figure supplement 2). Importantly, Wnt5a stimulation of NIH/3T3 cells in which Kif26b expression had been knocked out via CRISPR/Cas9-mediated genome editing resulted in no decrease in wound closure efficiency (i.e. had no effect on cell migration) (Figure 5—figure supplement 3), suggesting that Kif26b expression is required for Wnt5a-dependent changes in cell migration. Taken together, these results indicate that one key function of Kif26b is to promote cell migration, and that Wnt5a signaling may control the extent of cell migration by regulating the degradation of the Kif26b protein.

### In vivo perturbation of Kif26b function produces phenotypes characteristic of noncanonical Wnt5a-Ror signaling defects

We next sought to determine whether in vivo perturbation of Kif26b during embryonic development results in phenotypes that are consistent with an important function for Kif26b in Wnt5a-Ror signaling during embryonic development. A previously used approach for determining if a given signaling molecule functions as part of the noncanonical Wnt pathway was to determine if mis-expression of the signaling molecule leads to a phenotype similar to that observed when Wnt5a or Rors are mis-expressed in developing *Xenopus* or zebrafish embryos (Moon et al., 1993; Hikasa et al., 2002; Bai et al., 2014; Habas et al., 2001). Mis-expression of either Wnt5a or Ror2 in these embryos produces tissue morphogenesis defects, including defective convergent extension movements and a shortened or bent body axis, which are characteristic of disrupted noncanonical Wnt signaling. We therefore asked whether mis-expression of Kif26b protein in developing zebrafish embryos similarly induced phenotypes typical of abnormal noncanonical Wnt signaling.

**Figure 6.**
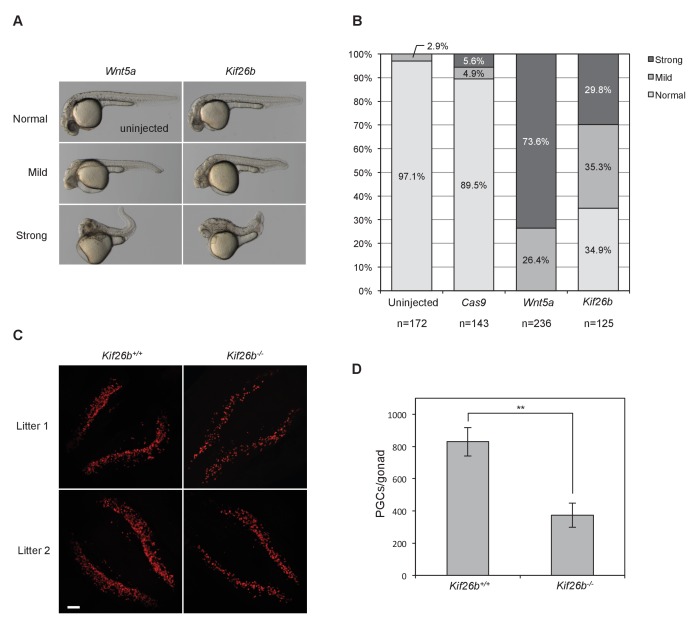
In vivo perturbation of Kif26b expression in zebrafish and mouse embryos induces phenotypes characteristic of Wnt5a-Ror signaling defects. (**A**) Representative images showing the effects of Wnt5a and Kif26b mis-expression on zebrafish embryonic tissue morphogenesis. Microinjection of *Wnt5a* mRNA did not produce any embryos that were scored as ‘normal’, so the image of an uninjected embryo is shown to represent normal embryos. (**B**) Quantification of the effects of Wnt5a and Kif26b mis-expression on zebrafish embryonic tissue morphogenesis. Images were taken from embryos at 50 hr post fertilization (50 hpf). Data for each experimental condition are pooled from at least three independent injection experiments. (**C**) Representative images showing immunofluorescence staining of SEAA1 to mark PGCs that have successfully entered the gonads of E11.5 *Kif26b^+/+^* or *Kif26b^-/-^* mouse embryos. Scale bar represents 100 μm. (**D**) Quantification of the numbers of PGCs per gonad in E11.5 *Kif26b^+/+^* or *Kif26b^-/-^* mouse embryos. Error bars represent ± SD calculated from independent biological samples (*Kif26b^+/+^*, n = 2; *Kif26b^-/-^*, n = 4). t-test (unpaired) was determined for *Kif26b^+/+^* vs. *Kif26b^-/-^* (*p*<0.01). 10.7554/eLife.26509.026Figure 6—source data 1.(Related to panel B) The effects of Wnt5a and Kif26b mis-expression on zebrafish embryonic tissue morphogenesis. 10.7554/eLife.26509.027Figure 6—source data 2.(Related to panel D) Quantification of the numbers of PGCs per gonad in E11.5 *Kif26b^+/+^* or *Kif26b^-/-^* mouse embryos.

To mis-express proteins in the developing zebrafish embryo, we microinjected in vitro-transcribed mRNA into one-cell stage embryos. Replicating previous reports (Moon et al., 1993; Bai et al., 2014), microinjection of *Wnt5a* mRNA caused axis truncation and bending phenotypes (Figure 6A,B). Strikingly, microinjection of *Kif26b* mRNA into one-cell zebrafish embryos also caused axis truncation and bending phenotypes resembling those caused by mis-expression of Wnt5a (Figure 6A,B). These phenotypes were rarely observed in uninjected embryos or in embryos injected with a negative control mRNA of similar size, *Cas9*, indicating that the axis truncation and bending phenotypes produced by *Kif26b* mis-expression are specific (Figure 6A,B). Together, these findings suggest that Kif26b specifically affects morphogenetic movements of cells in developing embryos in a manner similar to that of Wnt5a or Ror2 and support a model whereby Kif26b functions as part of a noncanonical Wnt5a-Ror regulatory cassette that regulates morphogenetic movements during embryogenesis.

To investigate further if Kif26b functions as part of the Wnt5a-Ror pathway during mouse development, we analyzed the development of primordial germ cells (PGCs), a process previously shown to require the Wnt5a-Ror pathway (Laird et al., 2011; Chawengsaksophak et al., 2012). During mouse embryogenesis, PGCs are specified from the epiblast at ~E7.25 and subsequently migrate through the hindgut and dorsal mesentery to populate the gonadal ridges by E11.5. PGCs that fail to enter the gonad are eliminated by programmed cell death (McLaren, 2003; Laird et al., 2008). Loss-of-function mutations in *Wnt5a* or *Ror2* alleles result in a substantial decrease in the number of PGCs that successfully colonize the gonad at E11.5 as compared to wild-type controls (~75% fewer in *Wnt5a* and ~50% fewer in *Ror2* mutants) (Chawengsaksophak et al., 2012; Laird et al., 2011), indicating that Wnt5a-Ror signaling is required for the proper colonization of the gonadal ridges by migrating PGCs. We reasoned that if Kif26b mediates biological activities of the Wnt5a-Ror pathway during PGC development, genetic perturbation of *Kif26b* might also disrupt PGC colonization of the gonads.

To test this hypothesis, we quantified the number of PGCs in the gonadal ridges of E11.5 *Kif26b^-/-^* embryos by whole-mount SSEA1 (a marker of PGCs) staining. The mean number of SSEA1-positive PGCs in the *Kif26b^-/-^* gonads (372.8 ± 74.95, n=4) was decreased significantly to 44.9% of *Kif26b^+/+^* littermate controls (829.5 ± 88.553, n=2) (Figure 6C,D). The relative similarity of the PGC depletion phenotypes observed in E11.5 *Kif26b^-/-^*, *Ror2^-/-^* and *Wnt5a^-/-^* mouse embryos suggest that Kif26b functions in a common signaling pathway with Wnt5a and Ror proteins to orchestrate PGC development. This experiment, taken together with the mis-expression analysis in zebrafish, provides in vivo evidence that Kif26b contributes to Wnt5a-Ror signaling in multiple tissues during embryonic development.

## Discussion

Since the seminal discovery that certain Wnt proteins can signal independently of β-catenin-mediated transcription to affect organ and tissue morphogenesis during development (Moon et al., 1993), few downstream effectors of this signaling system have been identified and validated in biological systems. In our previous work, we used in vivo mouse genetics to demonstrate that Ror receptors are essential mediators of a core noncanonical Wnt5a pathway crucial for tissue morphogenesis (Ho et al., 2012). In the current study, we integrate conditional mouse genetics with quantitative proteomics to identify new targets of Wnt5a-Ror signaling, and focus on one particularly high confidence target, Kif26b.

The first indication that Kif26b may mediate Wnt5a/Ror-dependent developmental processes came from studies in *C. elegans*. Orthologs of *Kif26b* (*vab-8*) and *Ror* (*cam-1*) were among the fourteen genes identified in a forward mutagenesis screen for genes required for the directional migration of the *C. elegans* canal-associated neuron (Forrester et al., 1998). *C. elegans* mutants of *vab-8* and *cam-1* display similar polarized cell migration and axon guidance defects where cell bodies and axons that normally move specifically toward the posterior end of the body become abnormally anteriorly displaced (Wightman et al., 1996; Forrester et al., 1999). In addition, both *vab-8* and *cam-1* mutants exhibit a lower penetrance withered-tail (Wit) phenotype reminiscent of the posterior A-P body axis truncation phenotype seen in *Wnt5a* or *Ror* mutant mice (Forrester et al., 1998). These studies, taken together with the findings in mammalian cells described in this article, suggest that Wnt5a, Ror and Kif26b proteins may function as part of an evolutionarily conserved pathway that orchestrates morphogenetic processes during the development of tissues.

Further evidence suggesting Kif26b may function in noncanonical Wnt signaling came from a recent study that identified Kif26b as a binding partner of Dvl3 in HUVECs (Guillabert-Gourgues et al., 2016), as Dvl proteins are known targets of Wnt5a-Ror signaling (Ho et al., 2012). Interestingly, this study demonstrated that Kif26b promotes the directional cell polarization and growth of HUVECs (Guillabert-Gourgues et al., 2016), consistent with our finding that Kif26b controls the migration of cells.

Our study demonstrates that Wnt5a-Ror signaling regulates Kif26b degradation, thereby influencing dynamic cellular processes such as the migratory behavior of cells. Moreover, by perturbing Kif26b function during embryogenesis, we provide in vivo evidence that Kif26b mediates certain biological effects of the Wnt5a-Ror pathway in body axis elongation and PGC development. Together, these findings suggest that a Wnt5a-Ror-Kif26b pathway comprises a conserved signaling cassette crucial for the execution of noncanonical Wnt functions.

### Biochemical mechanisms of Wnt5a-Ror-Kif26b signaling

**Figure 7.**
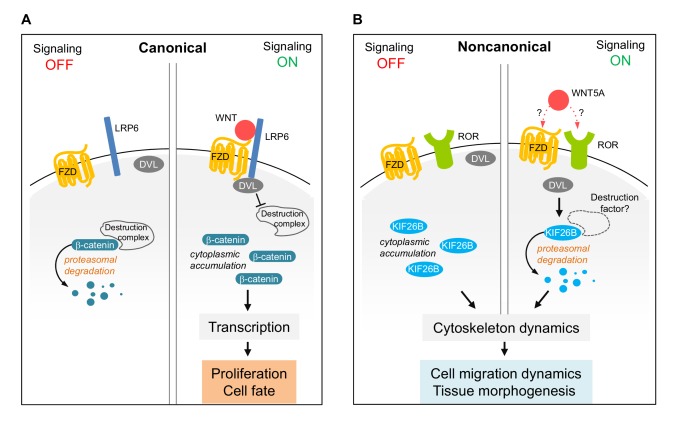
Model of Wnt5a-Ror-Kif26b signaling. Schematic of canonical Wnt/β-catenin signaling (**A**) vs. noncanonical Wnt5a-Ror-Kif26b signaling (**B**). See the main text for details.

Our observation that Wnt5a-Ror signaling triggers the ubiquitin/proteasome-dependent degradation of Kif26b demonstrates that UPS-mediated proteolysis is a conserved strategy employed by both the canonical Wnt/β-catenin and the noncanonical Wnt5a-Ror pathways to control the cellular abundance of their respective downstream effectors (Figure 7). In addition, we provide further evidence that Fzd and Dvl proteins are shared functional components of both Wnt signaling branches. Collectively, these observations suggest that the Wnt-Fzd-Dvl signaling module is an ancient and conserved feature common to multiple Wnt signaling systems. During evolution of the pathway, different signaling branches appear to have adopted additional regulatory mechanisms to achieve signaling and functional specificity. For example, while the Wnt/β-catenin pathway uses the Lrp5/6 receptors in conjunction with Fzd receptors to transmit a canonical Wnt signal across the plasma membrane, the Wnt5a-Ror pathway uses Ror1/2 receptors, likely also in conjunction with Fzds, to transmit a noncanonical Wnt signal.

At present, we do not understand the biochemical mechanisms by which Ror and/or Fzd receptors function in the pathway. In the conventional paradigm of receptor tyrosine kinase signaling, ligand binding induces receptor dimerization or oligomerization, which in turn enhances the intrinsic kinase activity of the receptor (Lemmon and Schlessinger, 2010). This then triggers receptor autophosphorylation and the subsequent recruitment and/or phosphorylation of downstream cellular effectors. Whether the Ror family of receptor tyrosine kinases possesses catalytically active kinase activity, however, is still under debate. It was reported that Wnt5a can stimulate the ability of full-length Ror2 to phosphorylate a GST-Ror2 kinase domain fusion protein on tyrosine residue(s) in vitro, and that mutagenesis of conserved tyrosine residues within the tyrosine kinase regulatory loop of Ror2 impairs the protein’s ability to repress canonical Wnt/β-catenin signaling (Mikels et al., 2009). Ror1 and Ror2, however, are known to harbor substitutions at amino acid residues generally believed to be critical for normal kinase function, and recent biochemical and structural studies have further suggested that the Ror proteins are pseudokinases (Gentile et al., 2011; Artim et al., 2012; Mendrola et al., 2013). In our phosphoproteomic study, we identified nine distinct Kif26b phosphorylation sites on eight unique phosphopeptides, and all these sites mapped to serine or threonine residues (Figure 1—figure supplement 2 and Supplementary file 1). In general, tyrosine phosphorylation is more dynamic than serine or threonine phosphorylation and tends to be underrepresented in large-scale phosphoproteomic studies (Lombardi et al., 2015). We therefore examined whether Wnt5a-Ror signaling induces tyrosine phosphorylation of Kif26b in NIH/3T3 cells using an anti-phosphotyrosine antibody-based affinity pull-down approach. However, under the various experimental conditions tested, we observed no effect of Wnt5a-Ror signaling on the tyrosine phosphorylation of Kif26b (S.S.C. and H.H.H., unpublished data). Thus, whether Ror receptors are active kinases or pseudokinases, and whether Kif26b is a direct substrate of these receptor tyrosine kinases will require further investigation.

It is also possible that phosphorylation of Kif26b by other cellular kinases is involved in Wnt5a-dependent regulation of Kif26b. Interestingly, a previous study showed that cyclin-dependent kinase (CDK) phosphorylates Kif26b on multiple serine and threonine sites and that these phosphorylation events play a critical role in controlling the stability of Kif26b by recruiting the E3 ubiquitin ligase Nedd4 (Terabayashi et al., 2012). It will be important in future studies to test whether these mechanisms, as well as the additional phosphorylation sites identified in our proteomic screens, are required for Wnt5a regulation of Kif26b degradation.

The functional interplay between Ror and Fzd receptors is also currently unclear. We have demonstrated that overexpression of Fzd family members is sufficient to induce Kif26b degradation in NIH/3T3 cells (Figure 4C,D). Overexpression of Ror2, however, does not have the same effect (E.P.K. and H.H.H., unpublished observation). These observations raise the possibility that Fzd proteins may function as the signaling receptors, while Ror proteins may play a modulatory role. This model is also consistent with our observation that even though endogenously expressed Wnt5a requires Ror1 and Ror2 expression to downregulate levels of Kif26b (Figures 1B and 2A), high concentrations of exogenously added Wnt5a can induce Kif26b degradation in Ror1 and Ror2 double knockout MEFs (M.W.S., M.E.G. and H.H.H., unpublished data), suggesting that Wnt5a can signal through receptors other than Rors. A deeper understanding of how Ror and Fzd proteins mediate Wnt5a-Kif26b signaling will require additional biochemical, structural and functional studies.

Our study also directly implicates Dvl proteins in promoting Kif26b degradation (Figure 4E,F) and further suggests a broader role for Dvl in regulating the stability of Wnt signaling effectors. Interestingly, during canonical Wnt signaling, Dvl functions to *inhibit* the degradation of β-catenin, whereas during noncanonical Wnt5a signaling, Dvl functions to *induce* the degradation of Kif26b (Figure 7). How Dvl transmits pathway-specific signals remains to be elucidated, but it likely involves differential post-translational modifications such as phosphorylation, differential protein-protein interactions, or both (Wallingford and Habas, 2005). The identification of Kif26b as a specific target of Dvl in the noncanonical Wnt signaling branch provides a new inroad to address this important question.

There is also emerging evidence that noncanonical Wnt signaling may function more generally to regulate the degradation of multiple proteins. For instance, Wnt5a signaling can induce the degradation of the cell adhesion receptor Syndecan 4 via the proteasome (Carvallo et al., 2010), and noncanonical Wnt5a and Wnt11 signaling has been associated with the proteasomal degradation of the focal adhesion molecule Paxillin (Kurayoshi et al., 2006; Iioka et al., 2007). It is possible that Wnt5a downregulates multiple effector molecules to affect the shape and behavior of cells in a variety of cell types and developmental contexts.

### Cellular and developmental functions of Kif26b

Data from our wound-healing assay suggest that one function of the Wnt5a-Ror-Kif26b axis is to modulate the migration of cells via Wnt5a-dependent degradation of Kif26b. How might Kif26b function at a subcellular level to affect cell migration? Mesenchymal cell migration typically involves four reiterative steps: protrusion of the leading edge, formation and maturation of focal adhesions, forward translocation of the cell body and finally retraction and de-adhesion of the trailing edge. Using the GFP-Kif26b NIH/3T3 cell line, we observed that the Kif26b protein is predominantly localized to the trailing edge of the cell, which is most consistent with a role of Kif26b in promoting trailing edge de-adhesion and/or retraction (Figure 3—figure supplement 2D,E; Video 1). This model is supported by a previously reported binding interaction between Kif26b and Myosin IIb, a key regulator of cell trailing edge de-adhesion and contractility (Uchiyama et al., 2010). It may also explain why Wnt5a has been reported in some studies to promote, while in others to suppress, cell migration (Kremenevskaja et al., 2005; Dissanayake et al., 2007; Nomachi et al., 2008; Enomoto et al., 2009; McDonald and Silver, 2009; Jiang et al., 2013; Liu et al., 2014; Zhang et al., 2014; Prasad et al., 2016; Yu et al., 2016; Connacher et al., 2017). It is well established that a delicate balance of cell adhesive and contractile activities dictates the manner by which cells migrate (Parsons et al., 2010; Huttenlocher and Horwitz, 2011). Moreover, this balance is likely to be different in different cell types and subject to further regulation by the extracellular environments to which the cells are exposed. Thus, if the primary function of the Wnt5a-Kif26b signaling axis is to modulate the strength of cell adhesion and/or contractility at the trailing edge, it could potentially manifest in opposing cell migratory behaviors depending on the cell types and the culture conditions used in a given study. Higher resolution imaging studies capable of directly measuring cell adhesion dynamics and contractile forces will be crucial to understanding how Kif26b functions at the trailing edge of the cell, or at other subcellular locations, to affect cell migration.

Although we have shown in a wound-healing assay that higher Kif26b levels promote the migration of NIH/3T3 cells and that Wnt5a signaling negatively regulates the migratory behavior of these cells via the degradation of Kif26b, it is likely that the signaling dynamics of the Wnt5a-Ror-Kif26b pathway are actually considerably more complex in vivo. During embryogenesis, migration and other morphogenetic processes must occur in a highly choreographed fashion where signals that promote and restrict cell movements are finely tuned in time and space. It is therefore possible that the relatively modest Wnt5a-dependent alteration of Kif26b levels we observed in vitro, as measured across entire cell populations, does not accurately reflect the dynamic regulation of the pathway that occurs during the development of tissues. The function of the Wnt5a-Ror pathway may not be simply to degrade Kif26b constitutively within a cell or tissue, but rather to tune the activity of Kif26b with a high degree of temporospatial resolution to achieve proper tissue morphogenesis. Indeed, cells are known to integrate and amplify shallow spatial gradients of extracellular cues into robust changes in behaviors, such as during axon pathfinding and neutrophil chemotaxis (von Philipsborn and Bastmeyer, 2007).

The in vivo significance of Kif26b in noncanonical Wnt5a-Ror function is demonstrated by our gene perturbation experiments. Disruption of Kif26b expression, whether by mis-expression in zebrafish or by loss-of-function gene ablation in mouse embryos, results in developmental defects that are similar to those observed when Wnt5a or Ror expression is perturbed. These findings, taken together with the observation that loss-of-function *cam-1* and *vab-8* mutants in *C. elegans* exhibit similar neuronal migration and polarization phenotypes (Forrester et al., 1998), support the model that Wnt5a, Ror and Kif26b comprise a signaling cassette crucial for the morphogenesis of tissues during development. However, it is important to note that while our in vivo experiments focus on comparing the gross morphological phenotypes of *Kif26b, Wnt5a and Ror* mutants, they do not directly demonstrate that the similarities in the observed phenotypes are caused by common underlying mechanisms. Therefore, it remains crucial to characterize the molecular and cellular basis of the *Wnt5a*, *Ror* and *Kif26b* mutant phenotypes, which will further establish the functional interactions among these molecules.

It is notable that genetic loss-of-function perturbation of Kif26b expression in mice does not closely mimic perturbation of Wnt5a or Ror expression in all developmental contexts. For instance, *Kif26b* knockout mice do not exhibit the global tissue truncation and craniofacial malformation phenotypes seen in *Wnt5a* and *Ror1/2* double mutants (Yamaguchi et al., 1999; Uchiyama et al., 2010; Ho et al., 2012). In addition, *Kif26b* knockout mice fail to elicit sprouting of the ureteric bud, resulting in kidney agenesis or severe kidney hypoplasia (Uchiyama et al., 2010), whereas *Wnt5a* or *Ror* knockout mice exhibit duplication of the ureteric bud, resulting in pleiotropic kidney defects (Huang et al., 2014; Nishita et al., 2014; Yun et al., 2014).

There are a number of explanations that might reconcile these seemingly discordant findings in different biological contexts. First, given the highly complex temporal and spatial dynamics that Wnt5a-Ror-Kif26b signaling likely undergoes in vivo, it becomes difficult to predict the ultimate phenotypes of embryos that constitutively lack the expression of downstream signaling components across development. Second, Kif26b may not be required in all tissues that undergo Wnt5a-Ror signaling during their morphogenesis. It is likely that other effector molecules may compensate for the genetic loss of *Kif26b* in certain developing tissues (Rossi et al., 2015). For instance, the closely related *Kif26a* gene is broadly expressed during mouse embryogenesis, and the Kif26a protein is also a target of Wnt5a-Ror signaling (M.K.S., E.P.K., H.H.H. unpublished data). Thus, one plausible explanation is that the developmental requirement of *Kif26b* in tissue extension and craniofacial morphogenesis may be masked by the continued expression of *Kif26a* in *Kif26b* knockout mice. By RT-qPCR, we verified that *Kif26a* mRNA is indeed expressed at appreciable levels in both wild-type and *Kif26b* knockout E12.5 MEFs, although we did not find evidence that *Kif26a* transcription is specifically upregulated in *Kif26b* knockout MEFs (E.P.K. and H.H.H., unpublished data). *Kif26a* single knockout mice also do not exhibit phenotypes typical of noncanonical Wnt5a-Ror signaling defects, instead manifesting with megacolon from enteric nerve hyperplasia (Zhou et al., 2009). Thus, discovering the full extent of developmental processes regulated by the Kinesin-11 family of proteins, as well as their functional relationship to the Wnt5a-Ror pathway, awaits a loss-of-function analysis of both *Kif26a* and *Kif26b* together, which may reveal non-overlapping phenotypes not present in either *Kif26a* or *Kif26b* single loss-of-function mutants. Lastly, it is also likely that other signaling branches apart from Kif26b exist within the Wnt5a-Ror pathway, and that these branches control distinct cellular processes that do not require the expression of the Kinesin-11 family of proteins. Thus, building a more complete inventory of the pathway components and understanding how these components interact functionally represents an important direction of future investigation.

## Materials and methods

### Mice

All mouse strains used in the study were described previously: *Ror1^f/f ^*(*Ho et al., 2012*), *Ror2^f/f^* (Ho et al., 2012), *CAG-CreER* (*Hayashi and McMahon, 2002*, stock # 004453, Jackson Laboratory, Bar Harbor, ME), *Wnt5a^-/- ^*(*Yamaguchi et al., 1999*, stock # 004758, Jackson Laboratory), and *Kif26b^f/f^* (Recuenco et al., 2015, stock # CDB0800K, RIKEN CDB, Japan, http://www2.clst.riken.jp/arg/mutant_list_file/CDB0800K.html). *Kif26b^-/-^* mice were produced by crossing *Kif26b^f/f^* mice to a ubiquitous Cre deleter line (*EIIA-Cre* [Lakso et al., 1996], stock # 003724, Jackson Laboratory). *Ror1^f/f^, Ror2^f/f^* and *CAG-CreER* mice were maintained in a mixed 129 and C57BL/6J background. *Wnt5a^-/-^ and Kif26b^-/-^ *mice were maintained in a C57BL/6J background. Wild-type mice are C57BL/6J. All animals were used according to institutional and NIH guidelines approved by the Institutional Animal Care and Use Committees at Harvard Medical School and University of California, Davis.

### Cell lines

Primary MEFs were isolated directly from mouse embryos as described (Ho et al., 2012) and used within three passages. NIH/3T3 Flp-In (R76107, Thermo Fisher Scientific, Hanover Park, IL) and HEK293T (CRL-3216, ATCC, Manassas, VA) cells were purchased and not re-authenticated. NIH/3T3 Flp-In and HEK293T cells were tested negative for mycoplasma contamination using the Universal Mycoplasma Detection Kit (30–1012K, ATCC). All cell lines were cultured at 37°C and 5% CO_2_ in Dulbecco’s Modified Eagles Medium (MT15017CV, Corning, Tewksbury, MA) supplemented with 1x glutamine (25–005 CI, Corning), 1x penicillin-streptomycin (30–002 CI, Corning) and 10% fetal bovine serum (16000069, Thermo Fisher Scientific).

### TMT/MS3 phosphoproteomic screen

Two E12.5 *Ror1^f/f^; Ror2^f/f^; CAG-CreER/+* embryos were individually dissected and used to derive primary MEFs. One E12.5 *Ror1^+/+^; Ror2^+/+^; CAG-CreER/+* embryo was used to derive the control MEFs. The primary passage of cells derived from each embryo was cultured in a 10-cm plate until confluent. The primary passage was then split into two 10 cm plates at six million cells per plate (day 0). 24 hr later (day 1), 4-OHT was added to one plate (0.25 μM final concentration), and the drug vehicle (EtOH) was added to the other plate. On day 2, a media change was performed using fresh media containing 0.25 μM 4-OHT. On day 3, a media change was performed again, but the final 4-OHT concentration was reduced to 0.1 μM. On day 4, a media change was performed without 4-OHT. On day 5, cells were washed once in ice-cold PBS, and cells from each 10-cm plate was scraped into 1 mL of ice-cold lysis buffer (8 M urea, 75 mM NaCl, 50 mM Tris pH 8.2, 1 mM NaF, 1 mM β-glycerophosphate, 1 mM Na_3_VO_4_, 10 mM Na_4_P_2_O_7_, 1 mM PMSF, and cOmplete protease inhibitor (-EDTA, 04693159001, Roche, Indianapolis, IN). Cells were homogenized by pipetting up and down using a P-1000 pipettor and then sonicated in a Bioruptor (Diagenode, Denville, NJ; 17 × 30 s ON/OFF cycles). Cell lysates were then centrifuged at 40,000 RPM for 20 min at 4°C. The clarified high-speed supernatants were collected, snap frozen in liquid nitrogen and stored at −80°C until the TMT/MS3 analysis was performed. Protein concentrations were determined using BCA reagents (23225, Thermo Fisher Scientific).

To perform the TMT/MS3 screen, tryptic peptides were prepared from whole cell lysates and the peptide mixtures from the six experimental conditions were individually labeled with the TMT reagents, such that reporter ions at m/z of 126, 127, 128, 129, 130 and 131 would be generated in the tandem spectrometry. Phosphopeptides were enriched by TiO_2_ chromatography. Liquid chromatography, MS3 tandem mass spectrometry and data analysis were carried out as previously described (Ting et al., 2011; McAlister et al., 2014; Paulo et al., 2015).

### Cloning of mouse *Kif26b cDNA*

A first-strand cDNA library was generated from MEF total RNA using M-MuLV reverse transcriptase (M0253S, New England BioLabs, Ipswich, MA) and the d(T)23VN primer according to the manufacturer’s instructions. This cDNA library was then used as template for PCR amplification of the *Kif26b* open reading frame with the following primers: Forward: gatcggccggcctaccatgaattcggtagccggaaataaag; Reverse: gatcggcgcgccttatcggcgcctggaggtgatgtc. The PCR product was subcloned into a modified pCS2+ vector using the FseI and AscI restriction sites. The entire *Kif26b* open reading frame was confirmed by sequencing.

### Antibodies

Antibodies against Kif26b were generated using a previously described antigen (Uchiyama et al., 2010). A C-terminal fragment of *Kif26b* was PCR amplified using the following primers and subcloned into a modified pGEX (28-9546-63, GE Healthcare, Pittsburgh, PA) vector to generate a GST fusion protein in *E. coli*. Forward: gatcggccggcctaccatgcgaaacgtgcaagagcctgagtcc; Reverse: gatcggcgcgccttatcggcgcctggaggtgatgtc. Protein expression was induced in the *E. coli* strain BL21(DE3) using IPTG (0.3 mM). To purify the C-terminal Kif26b protein fragment, bacterial pellets were lysed in STE (150 mM NaCl, 1 mM Tris pH 8.0, 1 mM EDTA) supplemented with protease inhibitors (04693159001, Roche) and 0.1 mg/ml lysozyme (L-6876, Sigma-Aldrich, St. Louis, MO) and incubated on ice for 15 min. Just before sonication, DTT was added to a final concentration of 2 mM and pre-diluted sodium lauroyl sarcosinate (in STE) was added to a final concentration of 10%. Lysates were then sonicated in a Bioruptor (5 × 30 s ON/OFF cycles) and then centrifuged at 60,000 RPM for 30 min at 4°C. Triton X-100 was added to the supernatant to a final concentration of 3% and incubated with Glutathione Sepharose 4B beads (17075601, GE Healthcare) for affinity purification. Purified C-terminal Kif26b proteins were dialyzed in PBS and used for immunization of rabbits.

Antibodies against Ror1 and Ror2 were described previously (Ho et al., 2012). The following antibodies were purchased: rabbit anti-Dvl2 (3216, Cell Signaling Technology, Danvers, MA); rabbit anti-EGFR (SC-03, Santa Cruz Biotechnology, Dallas, TX); goat anti-Wnt5A (AF645, R&D Systems, Minneapolis, MN); mouse anti-α-tubulin (clone DM1A, ab7291, Abcam, Cambridge, MA); chicken anti-GFP (GFP-1020, Aves Labs, Tigard, OR); rabbit anti-Myosin IIb (3404, Cell Signaling Technology); mouse anti-GM130 (610822, BD Biosciences, San Jose, CA); rabbit anti-phospho-LRP6 (Ser1490) (2568, Cell Signaling Technology).

### Western blotting

Quantitative western blotting was performed using the Odyssey infrared imaging system (Li-Cor Biosciences, Lincoln, IL) according to the manufacturer’s instructions. Non-saturated protein bands were quantified using Odyssey software, with the gamma level set at 1. Protein lysates for SDS-PAGE and western blotting were prepared in 1x or 2x LDS sample buffer (NP0008, Thermo Fisher Scientific) supplemented with 2-mercaptoethanol (5% final concentration). If BCA assays were required to quantify the protein lysate concentrations, the lysates were prepared instead in a homemade 1x SDS sample buffer (50 mM Tris pH 6.8, 2% SDS, 10% glycerol) without bromophenol blue or 2-mercaptoethanol. Once the protein concentrations were determined and normalized, the lysates were then mixed with 1/3 volume of 4x SDS sample buffer containing bromophenol blue (0.025%) and 2-mercaptoethanol (20%). Protein lysates used for Kif26b western blotting were not heated, as the Kif26b signal weakens substantially after heating, likely due to heat-induced protein aggregation. All other protein lysates were heated at 90°C for 5 min before SDS-PAGE and western blotting.

### Generation of shRNA targeting Kif26b

The lentiviral vector pLLX3.7 was used to generate recombinant lentiviruses expressing shRNA that target mouse *Kif26b*. The following sequences were targeted: gtgccttgcaaatctttat and gctcgagatacctcagaat.

### Generation of stable NIH/3T3 cell lines

To construct the GFP-Kif26b expression plasmid, the *eGFP* open reading frame was first subcloned into pENTR-2B (11816-014, Thermo Fisher Scientific), and the full-length mouse *Kif26b* open reading frame was subcloned in frame to the C-terminus of GFP. The resulting construct was verified by sequencing and then recombined with the pEF5-FRT-V5 vector (V602020, Thermo Fisher Scientific) using LR clonase II (11791100, Thermo Fisher Scientific) to create pEF5-GFP-Kif26b-FRT. The pEF5-GFP-Kif26b-FRT plasmid was used to generate stable isogenic cell lines using the Flp-In system and Flp-In NIH/3T3 cell line (Thermo Fisher Scientific). DNA transfection was performed in 10-cm plates with Genjet In Vitro Transfection Reagent (SL100488; SignaGen Laboratories, Rockville, MD). Cells that stably integrated the Flp-In constructs were selected using 200 μg/ml hygromycin B and expanded. A more detailed protocol is described at Bio-Protocol (Karuna et al., 2018).

### Lentivirus-mediated protein overexpression

Recombinant lentiviruses were generated using the pLEX_307 (for Dvl1 and all Fzd constructs) or pLVX-EF1α-mCherry-N1 (for Shisa2) vectors. Both vector systems use the EF1 promoter for driving transgene expression. pLEX_307 was a gift from David Root (Plasmid 41392, Addgene, Cambridge, MA) and pLVX-EF1α-mCherry-N1 was purchased (631986, Clontech Laboratories, Mountain View, CA). The human *Dvl1* open-reading frame was cloned by PCR from a HeLa cell cDNA pool. The mouse *Shisa2* open reading frame was PCR amplified from a *Shisa2*-containing plasmid (a gift from Xi He). The *Fzd* open reading frames were PCR amplified from the following Addgene plasmids: 42253, 42259, 42255, 42256, 42267, 42258, 42270 and 42261 (gifts from Chris Garcia and Jeremy Nathans). The open-reading frames in all lentiviral constructs were verified by sequencing. Lentiviruses were packaged and produced in HEK293T cells by co-transfection of the lentiviral vectors with the following packaging plasmids: pRSV-REV, pMD-2-G and pMD-Lg1-pRRE (gifts from Thomas Vierbuchen). 3 ml or 0.3 ml of the viral supernatants was used to infect WRK reporter cells seeded at 50% confluency in six-well plates. Puromycin selection (0.002 mg/ml) was carried out for 3 days. Cells from the viral titer that killed a large proportion of cells (60–90%) were expanded and used for FACS; this ensured that the multiplicity of infection (MOI) is ~1 for all cell lines used in the experiments.

### Generation of Kif26b knockout NIH/3T3 cell lines

The mouse *Kif26b* gene was mutated by CRIPSR/Cas9-mediated genome editing as according to (Ran et al., 2013). Briefly, LentiCRISPR V2 (a gift of Feng Zhang, Addgene plasmid 52961) was used to generate lentiviruses expressing small guide RNAs (sgRNAs). The sgRNA target sequences are: GCTTACGAGGAGTCGCGCGCCGG; GAACTGTAACGCCCGCTTGGTGG.

Following lentivirus infection and puromycin selection, NIH/3T3 cells were passaged for 3 days to allow time for mutagenesis to occur. Individual cell clones were picked from cell populations targeted with each of these sgRNAs, expanded and then screened initially by western blotting. Clones that appeared to lack Kif26b expression were sequenced to confirm genome modification.

### Cell proliferation and survival assays

For quantifications of cell proliferation, NIH/3T3 cell lines were plated on glass coverslips 24 hr prior to fixation. Cells were fixed with 4% paraformaldehyde in Cytoskeleton Buffer with sucrose (10 mM MES pH 6.1, 138 mM KCl, 3 mM MgCl, 2 mM EGTA, 0.32 M sucrose) for 20 min at room temperature, permeabilized in TBS-0.5% Triton X-100 for 10 min, then rinsed 3x with TBS-0.1% Triton X-100. Cells were blocked in Antibody Diluting Solution (AbDil) (TBS-0.1% Triton X-100, 2% BSA, 0.1% sodium azide) for 30 min at room temperature, then incubated overnight at 4°C with 1:500 of rabbit anti-phospho-Histone H3 (Ser10, Mitosis Marker) (#3377, Cell Signaling Technology) diluted in AbDil, or a no primary antibody control. After five washes in TBS-0.1% Triton X-100, Alexa dye-conjugated secondary antibodies were added at 1:1000 in AbDil for 45 min at room temperature. After five washes in TBS-0.1% Triton X-100, coverslips were mounted in DAPI Fluoromount-G (0100–20, SouthernBiotech, Birmingham, AL). Images of cells were acquired using a 10x objective at equal exposure, and then analyzed for the presence of nuclear staining of the Mitosis Marker per DAPI-positive nuclei counted.

For quantifications of cell survival, NIH/3T3 cells lines were plated similarly as for the cell proliferation assays. TUNEL staining was performed according to the manufacturer's instructions (In Situ Cell Death Detection Kit, TMR red, 12156792910, Roche), including a DNase-positive control (M0303S, New England BioLabs). Images of cells were acquired using a 10x objective at equal exposure, and then analyzed for the presence of nuclear TUNEL staining per DAPI-positive nuclei counted.

### Kinetic wound-healing cell migration assay

Cells were plated on 96-well plates (Essen ImageLock, 4379, Essen Instruments, Ann Arbor, MI). Wnt-C59 (100 nM final concentration) was added to cells 24 hr prior to wound creation with a wound scratcher (Essen Instruments). For Wnt5a treatment, recombinant Wnt5a (100 ng/ml final concentration) was added immediately after creation of wounds. Wound confluence was monitored with Incucyte Live-Cell Imaging System and software (Essen Instruments). Wound closure was observed every 1–2 hr for 48–96 hr by comparing the mean relative wound density of at least four biological replicates in each experiment.

### Immunocytochemistry

NIH/3T3 cells were plated at low density and grown on glass coverslips for 24 hr. Cells were rinsed 1x with PBS, then fixed with either ice-cold methanol for 3 min or with 4% paraformaldehyde in Cytoskeleton Buffer with sucrose for 20 min at room temperature. Cells were then rinsed 3x in PBS, permeabilized in TBS-0.5% Triton X-100 for 10 min, then rinsed 3x with TBS-0.1% Triton X-100. Cells were blocked in AbDil for 30 min at room temperature. Primary antibodies were added at the following dilutions in AbDil overnight at 4°C: chicken anti-GFP at 1:1000, mouse anti-α-tubulin at 1:5000, anti-Myosin IIb at 1:200, and anti-GM130 at 1:50. After five washes in TBS-0.1% Triton X-100, Alexa dye-conjugated secondary antibodies were added at 1:1000 in AbDil for 45 min at room temperature. After five washes in TBS-0.1% Triton X-100, coverslips were mounted in DAPI Fluoromount-G.

### Recombinant proteins and inhibitors

The following recombinant proteins and drugs were purchased: human/mouse Wnt5a (654-WN-010, R&D Systems); mouse Wnt3a (1324-WN-010, R&D Systems); mouse Dkk-1 (5897-DK-010, R&D Systems); Wnt-C59 (C7641-2s; Cellagen Technology, San Diego, CA); epoxomicin (A2606, ApexBio, Houston, TX); PYR-41 (B1492, ApexBio); cycloheximide (C7698-1G, Sigma-Aldrich).

### Reverse transcription and qPCR

Total RNA was isolated from MEFs using the PureLink RNA Mini Kit (121830108A, Thermo Fisher Scientific) according to the manufacturer's instructions. Isolated RNA was treated with DNase I (recombinant, RNase-free; 4716728001, Roche) and a cDNA library was synthesized using the cDNA High Capacity cDNA Reverse Transcription Kit (4368814, Thermo Fisher Scientific). The cDNA was the source of input for qPCR, using 7900 HT FAST and SYBR Green reagents (4329001, Thermo Fisher Scientific). The following qPCR primer pairs were used: *mKif26b* forward, CAAGTACGAGTGGCTGATGAA; *mKif26b* reverse, GGACCTGCTCCAAGTCAAAT; *β-actin* forward, GCTTCTAGGCGGACTGTTACTGA; *β-actin* reverse, GCGCAAGTTAGGTTTTGTCAAA.

### Flow cytometry

NIH/3T3 cells were plated at a density of 0.09 million/well in a poly-D-lysine-coated 48-well plate. 24 hr after plating, the cells were incubated with 10 nM Wnt-C59 and allowed to reach confluency. 48 hr after plating, cells were stimulated with either Wnt proteins or an equivalent volume of the control buffer (PBS with 0.1% BSA and 0.5% (w/v) CHAPS) in the presence of 10 nM Wnt-C59 for 6 hr. Cells were then harvested, resuspended in PBS + 0.5% FBS and analyzed using a flow cytometer (FACScan with a 488 nm laser, Becton Dickinson, San Jose, CA). Raw data were acquired with CellQuest (Becton Dickinson) and processed in FlowJoX (FLOWJO, Ashland, OR). Processing entailed gating out dead cells, calculation of median fluorescence, percent change of medians, and overlay of histograms. Dose-response curves based on percent change were fitted in MATLAB with the doseResponse function (written by Richie Smith and publicly available on Matlab File Exchange, File ID # 33604). A more detailed protocol is described at Bio-Protocol (Karuna et al., 2018).

### Live imaging

0.05 million cells were plated in a collagen-coated 35-mm glass bottom plate (P35GCOL-0-10-C, MatTek Corp, Ashland, MA). After adhering to the plate, cells were incubated in culture media supplemented with 25 mM Hepes and 10 nM Wnt-C59 for 24 hr. Cells were stimulated with 200 ng/ml recombinant Wnt5a in the presence of 25 mM Hepes and 10 nM Wnt-C59. Cells were imaged every 10 min for 16 hr, with 500 ms exposure at 40x magnification.

### Zebrafish

Wild-type NHGRI-1 fish were bred and maintained using standard procedures (LaFave et al., 2014). Embryos were obtained by natural spawning and staged as described (Kimmel et al., 1995). All zebrafish works were approved by the Institutional Animal Care and Use Committee, Office of Animal Welfare Assurance, University of California, Davis.

In vitro transcribed capped RNAs were prepared using the mMessage mMachine RNA Synthesis Kit (AM1340, Thermo Fisher Scientific) and purified using the RNeasy Mini Kit (74104, Qiagen, Germantown, MD) following manufacturers' instructions.

Microinjection of RNA was performed as described (Jao et al., 2012). In brief, one-cell-stage embryos from wild-type zebrafish intercrosses were injected with the following amounts of in vitro transcribed RNA: *Cas9*, 400 pg; *Kif26b*, 400 pg; *Wnt5a*, 150 pg. Pipettes were pulled on a micropipette puller (Model P-97, Sutter Instruments, Novato, CA). Injections were performed with an air injection apparatus (Pneumatic MPPI-2 Pressure Injector, Eugene, OR). Injected volume (typically ~1 nl) was calibrated with a microruler.

### PGC analysis

For PGC analysis, E11.5 embryos were dissected from timed matings. E0.5 is defined as noon of the day when the vaginal plug is detected. To expose the gonadal ridges, the abdominal cavity was opened and the visceral organs removed. The embryos were then cut just anterior to the forelimbs and just posterior to the hindlimbs. The midsection containing the gonadal ridges were washed once in cold (−20°C) methanol:DMSO (4:1), and then stored in the same fixative solution at −20°C until analysis. Genotypes were determined by PCR.

For whole-mount immunofluorescence, fixed embryos were rehydrated and rocked at 4°C overnight in PBSMT (PBS with 2% nonfat dry milk and 0.5% Triton X-100) with antibodies to SSEA1 (mouse IgM, MC-480, Developmental Studies Hybridoma Bank, Iowa City, IA, 1:200). Three PBSMT washes were followed by overnight incubation with secondary antibodies (1:200) and Hoechst (1:1000) in PBSMT. Embryos were then washed three times in PBS, dehydrated in a series of 5 min washes in 50%, 70%, 95%, and two times in 100% ethanol while rocking in the dark, and cleared with methyl salicylate for imaging.

Confocal imaging was carried out at room temperature with a 10x dry objective on a Leica SP5 TCS microscope equipped with 405, 488, 543, 594, and 633 nm lasers. Use of the 10x objective typically required the addition of a 1.5x digital zoom for optimal visualization of PGCs for quantification. Files of 1024 × 1024 pixel images with 2–3 μm distance between z-stacks were captured by a scanner with maximal frame resolution and Leica acquisition software. PGCs were counted on Imaris imaging software (Bitplane, Belfast, UK) using the Spots module. Spots of 7 μm size in the SSEA1 channel were identified by the software and visually inspected to confirm accuracy. All measurements were exported to Excel (Microsoft, Redmond, WA) for calculations and statistical analyses.

## Additional files

Supplementary file 1. Phosphopeptides identified and quantified in the TMT/MS3 phosphoproteomic screen. Columns include: Uniprot protein identification number, gene symbol, protein description/name, phosphosite position, phosphosite motif, localization score, spectral counts, the normalized summed signal to noise for each of the six TMT (126 to 131) channels.

Supplementary file 2. Hits from the TMT/MS3 phosphoproteomic screen. (A) Upregulated phosphopeptides that scored as ‘hits’ as defined in the text. Phosphopeptides above the bold line are ‘hits’ scored using the 2-fold cutoff filter. Phosphopeptides below the bold line are those scored between the 1.5- and 2-fold cutoffs. Columns include: gene name, protein description, fold change (4-OHT/vehicle treated samples), the *p *value. (B) Downregulated phosphopeptides that scored as ‘hits’ as defined in the text. Phosphopeptides above the bold line are ‘hits’ scored using the 2-fold cutoff filter. Phosphopeptides below the bold line are those scored between the 1.5- and 2-fold cutoffs. Columns include: gene name, protein description, fold change (4-OHT/vehicle treated samples), the *p* value.

Transparent reporting form
