# Supplementary material for: Kinesin superfamily protein Kif26b links Wnt5a-Ror signaling to the control of cell and tissue behaviors in vertebrates
Source: eLife. 2017 Sep 8;6:e26509. doi: 10.7554/eLife.26509 (PMC5590807; doi:10.7554/eLife.26509)
Supplement: Peer review history

# Decision letter

**DOI:** 10.7554/eLife.26509.033

**Affiliations:** Johns Hopkins University School of MedicineUnited States

In the interests of transparency, eLife includes the editorial decision letter and accompanying author responses. A lightly edited version of the letter sent to the authors after peer review is shown, indicating the most substantive concerns; minor comments are not usually included.

Thank you for submitting your article "Kinesin superfamily protein Kif26b links Wnt5a-Ror signaling to the control of cell and tissue behaviors" for consideration by *eLife*. Your article has been reviewed by three peer reviewers, and the evaluation has been overseen by Jeremy Nathans as the Reviewing Editor and Jonathan Cooper as the Senior Editor. One of the three reviewers, Karl Willert, has agreed to share his identity.

The reviewers have discussed the reviews with one another and the Reviewing Editor has drafted this decision to help you prepare a revised submission.

Three experts reviewed your manuscript, and their assessments, together with my views (Reviewing Editor), form the basis of this letter. As you will see, all of the reviewers were impressed with the importance and novelty of your work.

I am including the three reviews at the end of this letter, as there are a variety of specific and useful suggestions in them. In discussions among the reviewers, one over-arching point is that the strengths and weaknesses of the in vivo assays need to be made clear. Also, is it possible to specifically look directly at tyrosine phosphorylation of Kif26b? Does Wnt5a lead to an increase of P-Tyr on Kif26b? Such an experiment will not establish a direct link between Ror kinase activity and Kif26b phosphorylation, but it would provide additional insight into the signaling mechanism.

*Reviewer #1:*

This submission from Susman and colleagues provides a very comprehensive story about the role of Kip26b as a key downstream effector of Wnt5a-Ror2 signaling. The work encompasses proteomic screens, cell-based analyses, evaluation of genetically engineered mice, and evaluation of function in a zebrafish model. The comprehensive nature of this analysis provides a high degree of confidence in the overall model presented. However, there are a few questions that should be addressed prior to a decision being made on the manuscript. The most important of these is the nature of Kif26b phosphorylation induced by Wnt5a via Ror1/2.

1) The work identifying Kif26b as a protein that is differentially phosphorylated (or differentially stabilized) in the absence of Ror1/2 is convincingly presented. However, more information should be provided on what residues are phosphorylated within Kif26b downstream of Ror1/2 activation. Are there phospho-tyrosine residues present in Kif26b that can be identified based on the proteomics screen? Or are the residues responsible phospho-serine or phospho-threonine? There is very little discussion of this in the manuscript and it would seem to be a critical point of information regarding Kif26b stability and the nature of the signal downstream of Ror1/2 and Dvl.

*Reviewer #2:*

This manuscript describes an elegant set of experiments demonstrating that Kif26b protein stability is regulated by non-canonical Wnt signaling. This is an important piece of work as it provides the first robust biochemical assay of non-canonical Wnt signaling, an assay that has been sorely lacking for many years. As such, this finding will potentially redefine the direction of this field of research, especially since this increased protein turnover in response to Wnt5a-Ror signaling will likely apply to other proteins. Furthermore, the GFP reporter protein developed and characterized here is a powerful tool to monitor non-canonical Wnt signaling activity. Overall, this is an incredibly important finding supported by convincing and clear data.

There are a number of points that I would like to see addressed by the authors listed here, not in order of priority:

The result that Wnt3a, just like Wnt5a, leads to a reduction in Kif26b protein levels (Figure 2F) is quite interesting and indicates that one Wnt, in this case "canonical" Wnt3a, can simultaneously activate both pathways. The authors use rDKK1 to block canonical Wnt signaling and this has no effect on the reduction of Kif26b. The authors should extend this experiment by showing that inhibition of canonical Wnt signaling downstream of the Wnt-receptor interaction similarly has no effect on this readout. This can be achieved with tankyrase inhibitors, such as IWR.

Figure 3—figure supplement 1A: This experiment shows that inhibition of endogenous Wnt protein secretion with the Porcupine inhibitor C59 leads to an increase in Kif26b, as one would expect for blocking all endogenous Wnt signaling. This is a nice experiment and it should be extended by showing that Porcn inhibition can be rescued with Wnt5a protein. Furthermore, this assay should allow the authors to further address the effect of "canonical" Wnt signaling on Kif26b protein levels; e.g. does Wnt3a, like Wnt5a, reduce Kif26b protein? Does GSK3 inhibition (or const. active β-catenin) reduce Kif26b levels? Such experiments would solidify the claim that the effect on Kif26b is independent of the well-studied Wnt/β-catenin pathway.

Figure 2—figure supplement 1A: it looks like shRNA-mediated KD of Kif26b leads to a reduction in Dvl2 phosphorylation. Is that the case? If yes, is there some sort of reciprocal interaction between Kif26b and Dvl-phosphorylation. Figure 3G and H further speak to this possibility. Could the authors address this possibility?

Figure 3—figure supplement 2A: It is unclear whether the protein detected here is WT Kif26b or the GFP-Kif26b fusion protein. Figure 4—figure supplement 1A suggests that the majority of detected protein is overexpressed fusion protein, however, can the authors verify this by probing this blot with GFP antibody to only detect the GFP reporter protein?

In regards to the GFP-Kif26b reporter: with this assay, the authors should be able to narrow down the domain of Kif26b that confers the destabilization effect on GFP. A series of truncations of Kif26b could identify the critical region and provide additional information about the mechanism by which this protein is targeted for degradation by Wnt5a-Ror signaling. Perhaps such experiments are intended for a future publication, but it certainly would be a logical extension that would substantially enhance this current study.

Figure 3—figure supplement 2B-E: Can the authors show GFP-Kif26b protein levels and localization upon Wnt5a stimulation? Does the signal diminish to undetectable levels? In a time-course treatment, does GFP signal retract from the processes highlighted in panel E?

Figure 4—figure supplement 1C: It looks like Mutant allele 2 produced with sgRNA2 represents a 13bp deletion that leads to a premature stop-codon: the authors should clarify this by depicting the deletion as shown for the mutant alleles produced with sgRNA1.

Figure 4: The authors show that reduction of Kif26b protein, either by KO or by Wnt5a treatment, leads to reduction in the migratory behavior of NIH3T3 cells. Although these data are quite clear, they contradict several prior studies that showed that Wnt5a treatment leads to an increase in cell migration (e.g. see Figure 7A in PMID: 24658098, Figure 3 in PMID: 17426020, Figure 1 in PMID: 24524196, to name a few). While this may be a context/cell type dependent effect of Wnt5a, the authors need to address this discrepancy.

*Reviewer #3:*

Wnt cell-cell signals regulate embryonic development and tissue homeostasis in virtually every tissue and organ. There thus is intense interest in their signaling pathways. The components of the canonical β-catenin pathway have largely been identified, but the components of the alternate Wnt signaling pathway mediated by Ror kinases are less clear. Here the authors take a proteomics approach to identify proteins whose abundance or phosphorylation are regulated by Ror kinase signaling. They identify the kinesin Kif26B as a protein whose abundance is negatively regulated by Wnt5a/Ror signaling. They provide clear evidence that this involves targeting it for destruction by the proteasome. They then add some data suggesting possible phenotypic consequences of loss or overexpression of Kif26B. These data provide some support for the idea that may be an effector of non-canonical Wnt signaling, but are less compelling. Overall, I thought the weaknesses significantly reduced my enthusiasm.

Strengths:

1) Strong interest in the pathway in the cell and developmental biology community.

2) Solid biochemical data in the first half of the paper.

Weaknesses:

1) The effect of the pathway on Kif26B stability, while clear, was relatively modest (50% reduction).

2) The biological backup testing the role of Kif26B in Wnt/Ror signaling was less compelling, involving either relatively superficial similarities after over-expression or partial effects after loss-of-function.

Major issues:

1) While the proteomic and biochemical data supporting downregulation of Kif26B by Wnt/Ror signaling were clear, the authors should have provided a quantitative assessment of the degree of downregulation-for example in Figure 2A it looks like the effect is only 2-fold but the loading control is very over-exposed so it's difficult to assess. This would have made it clearer that while Kif26B is destabilized by Wnt/Ror signaling, significant levels remain-this is important for determining biological consequences. Similarly, Wnt5a treatment of 3T3 cells also only led to a 50% reduction (Figure 3), and since Kif26B heterozygous mice are wild-type in phenotype, one wonders how critical this effect would be in vivo. Further, given the β-catenin paradigm, an effort to see if particular subcellular pools of Kif26B were preferentially targeted for destruction would have been revealing.

2) The loss- and gain-of-function assays were not very sophisticated or mechanistically compelling. The "cell migration assay" was presented simply as quantitation of cell density, with only a single image presented (I would also question whether those fine protrusions seen in that image are "retraction fibers) and without any further analysis of mechanism by which Kif26B might affect this process. The fact that Wnt5a can block the effects of Kif26B overexpression was rather surprising, given that over-expression looked to be 10 fold and Wn5a in other contexts only reduced levels by 50%. They also missed what I view as the best test in this cell line-does Kif26B KO block the effects of Wnt5a on migration. Finally, I would question the idea that NIH 3T3 cells, in culture for 70 years, represent a good model for "mouse embryonic mesenchymal cells that undergo morphogenetic movements during development". The contrast to the earlier work by others on Kif26B in HUVEC cells was striking-in that work the authors presented abundant cell biological data assessing the mechanisms by which Kif26B regulated cell migration. The overexpression assay in zebrafish was intriguing, but once again the authors only took a superficial look at the effects on body morphology without assessing underlying mechanisms. The sole in vivo loss-of-function assay involved a single pair of pictures revealing a modest reduction in primordial germ cell numbers.

3) The authors approach to the previous literature was a bit odd. Key information about what we knew already about Kif26B was either left out entirely or mentioned only in the Discussion. The known role for the *C. elegans* homolog in Ror signaling and the recent work demonstrating a role in planar polarity signaling in vivo in the mouse downstream of Dvl were highly relevant background information and should have been mentioned in the Introduction, as should the knockout phenotype of the gene in the mouse (kidney defects), as this puts limits on how general a role it has in Wnt5 signaling. They also should have reviewed work on phosophoregulation of ubiqitination of Kif26B (Terabayashi et al., 2012).

4) The authors, in my mind, overstate the case for Kif26B as a key player in Wnt/Ror signaling. The Kif26B knockout mouse has an interesting kidney phenotype, but appears to lack many of the phenotypes of the Wnt5a knockout, and Wnt5a is not the only Wnt with potential non-canonical signaling functions. These data suggest that, at best, Kif26B acts in a subset of tissues. The role of Kif26A, mentioned only in passing, also needs to be considered-it also has a known mouse KO phenotype, also one without a clear Wnt/Ror signaling connection.

# Reply: Author response

**DOI:** 10.7554/eLife.26509.034

*In discussions among the reviewers, one over-arching point is that the strengths and weaknesses of the* in vivo *assays need to be made clear. Also, is it possible to specifically look directly at tyrosine phosphorylation of Kif26b? Does Wnt5a lead to an increase of P-Tyr on Kif26b? Such an experiment will not establish a direct link between Ror kinase activity and Kif26b phosphorylation, but it would provide additional insight into the signaling mechanism.*

We have now explicitly pointed out the strengths and weaknesses of our in vivo assays in the Discussion. In particular, we emphasized that while our in vivo experiments focus on comparing the gross morphological phenotypes of Kif26b, Wnt5a and Ror mutants, they do not directly demonstrate that the similarities in the observed phenotypes are caused by common underlying mechanisms. In addition, we have devoted the last two paragraphs of the Discussion to consider the possible reasons behind the observation that the Kif26b single knockout mice do not fully phenocopy the tissue truncation defects seen the Wnt5a or Ror1/2 double mutants.

Regarding whether Wnt5a leads to an increase of P-Tyr on Kif26b, we have conducted new experiments to explore this possibility. However, under the various experimental conditions tested, we did not find evidence that activation of the Wnt5a-Ror pathway stimulates tyrosine phosphorylation of Kif26b. Consistent with this observation, all of the Kif26b phosphorylation sites identified in our proteomic screen mapped to either serine or threonine residues. We have added a new figure supplement (Figure 1—figure supplement 2) to more clearly show the Kif26b phosphorylation sites identified in the proteomic screen and included a new paragraph in Discussion to discuss these findings. We also discussed the current literature concerning whether the Ror receptors are active tyrosine kinases or pseudokinases.

Reviewer #1:

*This submission from Susman and colleagues provides a very comprehensive story about the role of Kip26b as a key downstream effector of Wnt5a-Ror2 signaling. The work encompasses proteomic screens, cell-based analyses, evaluation of genetically engineered mice, and evaluation of function in a zebrafish model. The comprehensive nature of this analysis provides a high degree of confidence in the overall model presented. However, there are a few questions that should be addressed prior to a decision being made on the manuscript. The most important of these is the nature of Kif26b phosphorylation induced by Wnt5a via Ror1/2.*

*1) The work identifying Kif26b as a protein that is differentially phosphorylated (or differentially stabilized) in the absence of Ror1/2 is convincingly presented. However, more information should be provided on what residues are phosphorylated within Kif26b downstream of Ror1/2 activation. Are there phospho-tyrosine residues present in Kif26b that can be identified based on the proteomics screen? Or are the residues responsible phospho-serine or phospho-threonine? There is very little discussion of this in the manuscript and it would seem to be a critical point of information regarding Kif26b stability and the nature of the signal downstream of Ror1/2 and Dvl.*

Please see our response under “Reviewing Editor’s Comments” above.

Reviewer #2:

*[…] The result that Wnt3a, just like Wnt5a, leads to a reduction in Kif26b protein levels (Figure 2F) is quite interesting and indicates that one Wnt, in this case "canonical" Wnt3a, can simultaneously activate both pathways. The authors use rDKK1 to block canonical Wnt signaling and this has no effect on the reduction of Kif26b. The authors should extend this experiment by showing that inhibition of canonical Wnt signaling downstream of the Wnt-receptor interaction similarly has no effect on this readout. This can be achieved with tankyrase inhibitors, such as IWR.*

We have examined the effect of the tankyrase inhibitor IWR-1 on Wnt3a- and Wnt5a-induced Kif26b downregulation. Consistent with Wnt3a and Wnt5a signaling independently of the Wnt/β-catenin pathway, IWR-1 treatment did not affect the ability of Wnt3a or Wnt5a to downregulate the expression of Kif26b in MEFs. This result is presented in Figure 2G of the revised manuscript.

*Figure 3—figure supplement 1A: This experiment shows that inhibition of endogenous Wnt protein secretion with the Porcupine inhibitor C59 leads to an increase in Kif26b, as one would expect for blocking all endogenous Wnt signaling. This is a nice experiment and it should be extended by showing that Porcn inhibition can be rescued with Wnt5a protein. Furthermore, this assay should allow the authors to further address the effect of "canonical" Wnt signaling on Kif26b protein levels; e.g. does Wnt3a, like Wnt5a, reduce Kif26b protein? Does GSK3 inhibition (or const. active β-catenin) reduce Kif26b levels? Such experiments would solidify the claim that the effect on Kif26b is independent of the well-studied Wnt/β-catenin pathway.*

We have repeated the C59 treatment experiment to additionally show that the effect of C59 on WT MEFs (i.e. C59 causes an increase in Kif26b expression in these cells) can be effectively reversed by the application of ectopic Wnt5a. This result is presented in Figure 3—figure supplement 1A of the revised manuscript. We did not further interrogate the roles of Wnt/β-catenin pathway components here, because the main purpose of this figure supplement is to demonstrate that NIH/3T3 cells can respond to Wnt5a stimulation in a similar manner as MEFs. We feel that with the addition of the new IWR-1 experiment presented in Figure 2G, there is sufficient evidence to support the model that Kif26b degradation occurs via a noncanonical Wnt signaling mechanism.

*Figure 2—figure supplement 1A: it looks like shRNA-mediated KD of Kif26b leads to a reduction in Dvl2 phosphorylation. Is that the case? If yes, is there some sort of reciprocal interaction between Kif26b and Dvl-phosphorylation. Figure 3G and H further speak to this possibility. Could the authors address this possibility?*

We have independently investigated this possibility by comparing the extent of Dvl2 phosphorylation in MEFs that were prepared from WT and Kif26b KO embryos. We observed the same levels of Dvl2 phosphorylation in WT and Kif26b MEFs, suggesting that Kif26b does not reciprocally regulate Dvl2 phosphorylation.

*Figure 3—figure supplement 2A: It is unclear whether the protein detected here is WT Kif26b or the GFP-Kif26b fusion protein. Figure 4—figure supplement 1A suggests that the majority of detected protein is overexpressed fusion protein, however, can the authors verify this by probing this blot with GFP antibody to only detect the GFP reporter protein?*

We have now included an anti-GFP blot in Figure 5—figure supplement 1A of the revised manuscript.

*In regards to the GFP-Kif26b reporter: with this assay, the authors should be able to narrow down the domain of Kif26b that confers the destabilization effect on GFP. A series of truncations of Kif26b could identify the critical region and provide additional information about the mechanism by which this protein is targeted for degradation by Wnt5a-Ror signaling. Perhaps such experiments are intended for a future publication, but it certainly would be a logical extension that would substantially enhance this current study.*

We agree with the Reviewer that the GFP-Kif26b reporter is a good tool to map the domain of Kif26b that mediates Wnt5a-induced Kif26b degradation. We are currently working on this project. However, we would prefer to publish this work in a separate, future study where we not only map the domain of Kif26b that mediates Wnt5a-depenent degradation, but also identify the specific amino acid residues and the detailed biochemical mechanisms involved in this regulation.

*Figure 3—figure supplement 2B-E: Can the authors show GFP-Kif26b protein levels and localization upon Wnt5a stimulation? Does the signal diminish to undetectable levels? In a time-course treatment, does GFP signal retract from the processes highlighted in panel E?*

We have included in the revised manuscript a movie of GFP-Kif26b-expressing NIH/3T3 cells without or with Wnt5a treatment (Video 1 of the revised manuscript). The movie shows that the fluorescent signals of the GFPKif26b fusion protein decrease robustly with Wnt5a stimulation in a time-dependent manner. The movie also shows that the processes similar to those highlighted in panel E of the revised Figure 3—figure supplement 2 do appear to retract rapidly as the cells migrate forward. However, with the resolution of the movie, it is difficult to discern whether the GFP-Kif26b signal retracts within these fine processes.

*Figure 4—figure supplement 1C: It looks like Mutant allele 2 produced with sgRNA2 represents a 13bp deletion that leads to a premature stop-codon: the authors should clarify this by depicting the deletion as shown for the mutant alleles produced with sgRNA1.*

We have revised the panel (Figure 5—figure supplement 1C of the revised manuscript) to better depict the mutation.

*Figure 4: The authors show that reduction of Kif26b protein, either by KO or by Wnt5a treatment, leads to reduction in the migratory behavior of NIH3T3 cells. Although these data are quite clear, they contradict several prior studies that showed that Wnt5a treatment leads to an increase in cell migration (e.g. see Figure 7A in PMID: 24658098, Figure 3 in PMID: 17426020, Figure 1 in PMID: 24524196, to name a few). While this may be a context/cell type dependent effect of Wnt5a, the authors need to address this discrepancy.*

We have addressed this discrepancy in the Discussion of the revised manuscript.

Reviewer #3:

*[…] Major issues:*

*1) While the proteomic and biochemical data supporting downregulation of Kif26B by Wnt/Ror signaling were clear, the authors should have provided a quantitative assessment of the degree of downregulation-for example in Figure 2A it looks like the effect is only 2-fold but the loading control is very over-exposed so it's difficult to assess. This would have made it clearer that while Kif26B is destabilized by Wnt/Ror signaling, significant levels remain-this is important for determining biological consequences. Similarly, Wnt5a treatment of 3T3 cells also only led to a 50% reduction (Figure 3), and since Kif26B heterozygous mice are wild-type in phenotype, one wonders how critical this effect would be* in vivo*. Further, given the β-catenin paradigm, an effort to see if particular subcellular pools of Kif26B were preferentially targeted for destruction would have been revealing.*

We have clarified in the Figure 2 legend that all immunoblot samples were normalized by BCA assays for total protein. The approximately 2-fold change in Kif26b levels as seen in the western blots is consistent with what we observed by flow cytometry using the GFP-Kif26b reporter cell line. However, these assays average the changes in Kif26b levels across the entire cell population. Thus, it remains possible that the actual changes are greater than 2-fold in specific subcellular locations or in the developing embryos. We have considered these possibilities in the Discussion of the revised manuscript.

The idea that particular subcellular pools of Kif26b may be preferentially targeted for degradation is interesting. However, in the video of GFP-Kif26b-expressing NIH/3T3 cells that we have added, we did not find evidence that a particular subcellular pool of Kif26b is preferentially degraded. Higher resolution imaging will be required to explore this possibility in future studies.

*2) The loss- and gain-of-function assays were not very sophisticated or mechanistically compelling. The "cell migration assay" was presented simply as quantitation of cell density, with only a single image presented (I would also question whether those fine protrusions seen in that image are "retraction fibers) and without any further analysis of mechanism by which Kif26B might affect this process. The fact that Wnt5a can block the effects of Kif26B overexpression was rather surprising, given that over-expression looked to be 10 fold and Wn5a in other contexts only reduced levels by 50%. They also missed what I view as the best test in this cell line-does Kif26B KO block the effects of Wnt5a on migration. Finally, I would question the idea that NIH 3T3 cells, in culture for 70 years, represent a good model for "mouse embryonic mesenchymal cells that undergo morphogenetic movements during development". The contrast to the earlier work by others on Kif26B in HUVEC cells was striking-in that work the authors presented abundant cell biological data assessing the mechanisms by which Kif26B regulated cell migration. The overexpression assay in zebrafish was intriguing, but once again the authors only took a superficial look at the effects on body morphology without assessing underlying mechanisms. The sole* in vivo *loss-of-function assay involved a single pair of pictures revealing a modest reduction in primordial germ cell numbers.*

We agree that it is important to develop a deeper understanding of the mechanisms by which Kif26b controls cell migration and embryonic development. However, given that our preliminary data indicated that the highly related Kif26a is also a regulatory target of the Wnt5a-Ror pathway, we feel that a more rigorous way to investigate the physiological functions of the Kif26 family is to analyze Kif26a/Kif26b double knockout animals, which is beyond the scope of this paper. In the revised manuscript, we have included additional discussion on the strengths and weaknesses of the in vivo models used in this study (please also see our response under “Reviewing Editor’s Comments”).

Regarding the effect of Wnt5a treatment on Kif26b knockout NIH/3T3 cells, we have now included data showing that Wnt5a does not further decrease the rate of wound closure in Kif26b knockout cells (Figure 5—figure supplement 3). We also emphasize in the revised manuscript that even though we use NIH/3T3 cells as an in vitro model for understanding Kif26b’s role in cell migration, the functions of Kif26b (and the Wnt5-Ror pathway) are likely to be much more complex in vivo and may involve processes and regulations beyond what we observed in NIH/3T3 cells.

*3) The authors approach to the previous literature was a bit odd. Key information about what we knew already about Kif26B was either left out entirely or mentioned only in the Discussion. The known role for the C. elegans homolog in Ror signaling and the recent work demonstrating a role in planar polarity signaling* in vivo *in the mouse downstream of Dvl were highly relevant background information and should have been mentioned in the Introduction, as should the knockout phenotype of the gene in the mouse (kidney defects), as this puts limits on how general a role it has in Wnt5 signaling. They also should have reviewed work on phosophoregulation of ubiqitination of Kif26B (Terabayashi et al., 2012).*

We have expanded the Introduction to include previous work on the *C. elegans* homolog of Kif26b, and more recent mouse studies on the role of Kif26b in cell adhesion, polarity and migration. We have also included additional discussion on the Kif26b mouse knockout phenotypes and offered possible explanations for the observation that the Kif26b mutant mice lack the global tissue morphogenesis phenotype previously seen in Wnt5a or Ror1/2 double mutant animals. We feel that this comparison of the mouse phenotypes is more appropriate in the Discussion. Finally, we have incorporated in the Discussion a review of the work of Terabayashi et al. on phosphoregulation of Kif26b ubiquitination.

4) The authors, in my mind, overstate the case for Kif26B as a key player in Wnt/Ror signaling. The Kif26B knockout mouse has an interesting kidney phenotype, but appears to lack many of the phenotypes of the Wnt5a knockout, and Wnt5a is not the only Wnt with potential non-canonical signaling functions. These data suggest that, at best, Kif26B acts in a subset of tissues. The role of Kif26A, mentioned only in passing, also needs to be considered-it also has a known mouse KO phenotype, also one without a clear Wnt/Ror signaling connection.

In the revised Discussion, we expanded our consideration of the possible role of Kif26a in Wnt5a-Ror signaling. Furthermore, we have also considered the possibility that other signaling branches apart from Kif26b exist within the Wnt5a-Ror pathway, and that these branches may function independently of the Kif26 family to mediate distinct cellular functions of the Wnt5a pathway.
